# Co-digestion of palm oil mill effluent with chicken manure and crude glycerol: biochemical methane potential by monod kinetics

**DOI:** 10.1016/j.heliyon.2021.e06204

**Published:** 2021-02-10

**Authors:** Narongsak Seekao, Sawinee Sangsri, Nirattisai Rakmak, Wipawee Dechapanya, Chairat Siripatana

**Affiliations:** a School of Engineering and Technology, Walailak University, 80161, Nakhon Si Thammarat, Thailand; b Biomass and Oil-Palm Excellence Center, Walailak University, 80161, Nakhon Si Thammarat, Thailand

**Keywords:** Palm oil mill effluent, Co-digestion, Multi-substrate monod model, Chicken manure, Crude glycerol, Kinetic of co-digestion, Biochemical methane potential

## Abstract

In Thailand, the palm oil industry produces a huge amount of palm oil mill effluent (POME), mostly used for electricity generation through biogas production. Co-digestion with other waste can further improve biogas yield and solve waste management problems. Most previous studies relied on biochemical methane potential (BMP) assay or batch co-digestion to obtain the optimal mixing ratio, ignoring the kinetic part or treat it for sole discussion of the results. This work directly uses mechanistic models based on Monod kinetics to describe the experimental results obtained from the co-digestion of POME (40 ml, BMP = 281.2 mlCH_4_/gCODadded)) with chicken manure (CM) (0–50 g) and crude glycerol (Gly) (0–10 ml). The best mixing ratio between CM and POME was 5 gCM: 40 mlPOME (BMP = 276.9 mlCH_4_/gCODadded). The best ratio for Gly and POME was 2 mlGly: 40 mlPOME (BMP = 211.9 mlCH_4_/gCODadded). Adding Gly only 2 mlGly/40 mlPOME doubled the amount of biogas. Hence, crude glycerol is a good substrate for on-demand biogas output. The co-digestion increases the methane output but with a decreased yield. A multi-substrate Monod model was developed based on the levels of digestion difficulty. A partial-least squared fitting was used to estimate its main parameters. All parameters included in the model passed the significant tests at a 95% confidence level. The model can describe the experimental results very well, predict observable state variables of batch co-digestion, and allow a simple extension for continuous co-digestion dynamics. A limited continuous experiment was conducted to confirm the applicability of the model parameters of POME digestion obtained from BMP tests to predict a continuous AD. The results show good potential but must be carefully interpreted. It is generally possible and practical to directly obtain design and operational parameters from BMP assays based on only accumulated biogas curves and initial and final COD/VS.

## 1 Introduction

### 1.1 Background

Thailand is the third-largest palm producer, following Malaysia and Indonesia. It is estimated that one tonne of crude palm oil produces 5–7.5 m^3^ of palm oil mill effluent (POME), of which more than 50% ends up in POME [1]. POME is the wastewater characterized by a thick-yellowish liquid, high organic contents, having chemical oxygen demand (COD) and biochemical oxygen demand (BOD) in the range of 44,300–102,692 mg/L and 25,000–65,714 mg/L, respectively. It is suitable for generating electricity through biogas. POME has low pH (pH 3.4–5.2) because it contains many organic acids produced by the initial fermentation process. It has a high concentration of total solid (40,500 mg l^−1^) and high suspended solid (18,000–46,011 mg/l). The concentration of oil and grease ranges from 4,000–9,341 mg/l, depending on milling processes [2, 3, 4, 5]. Characteristics of POME depends on the quality of the raw material (fresh palm-oil fruit bunches) and the efficiency of palm oil extraction processes [6].

POME has become a commodity rather than undesirable wastewater, providing energy cost savings and additional income for the palm oil mills (POMs). The main reasons for its suitability for anaerobic digestion are its high organic content, relatively easily digestible, and low toxicity to the working microorganisms. POME supply is dependable both in terms of quality and quantity. POME composition also falls into the optimal range of C/N ratio (20–30) [7, 8, 9]. It is rich in carbohydrates and having sufficient nitrogen-containing components (like protein and other nitrogen sources).

### 1.2 Modeling of AD batch co-digestion

Although biogas plant using POME is widely practiced, and much research is centered around POME as a substrate for biogas production, there are challenging problems that need to address. Firstly, most of the biochemical methane potential (BMP) data for various co-digestion types do not directly lend themselves to industrial applications. Most authors do not report design or operational parameters used for quantitative prediction other than those specific to the authors' experiments. Many authors use Gompertz or Logistic-type or other empirical models to interpret BMP data objectively, ensuring unbiased BMP results. A few works have tried to analyze and extract design and operational parameters using the Monod-type kinetics, but the approach has not yet been widely used [10, 11]. Secondly, it is well-established that the co-digestion of POME with various wastes can enhance the yield and productivity of biogas (CH_4_/H_2_) production. Still, there are only limited industrial applications because of logistic constraints, operational uncertainty and stability, and lack of suitable predictive tools. Typically, before commissioning co-digestion on an industrial scale, a full dynamic study in pilot-scale systems must be conducted to ensure long-term stability. Without proper modeling tools, BMP assay or batch co-digestion tests can only provide minimum process information.

However, BMP assay and batch co-digestion experiments have many advantages. It is efficient in terms of cost and time for operational-range screening and optimization, substrate, and microbial optimization. But unless done correctly, the results may not accurately reflect the intended measurement.

Recently, Koch et al. [12] discussed the power and limitation of BMP tests. The authors argued that the BMP tests could only give preliminary degradation kinetics due to the operational differences between BMP experiments and the continuously operated reactors. If hydrolysis steps limit the AD, it is well known that this step is slower in the BMP test than in continuous operation. The differences are also attributed to the high initial organic loading rate and the inoculum acclimation in the BMP test [13, 14]. They noted that one should not use BMP tests in identifying synergistic or antagonistic effects in AcoD.

BMP tests can not provide evidence of the substrate's chronic toxicity because of the high proportion of inoculum. Also, the substrate is fed only once at the beginning of the test. In general, the BMP test can not provide information on methane yield, process stability, and possible organic loading rate expected in a continuous system. This argument may be valid. However, it does not negate the importance of modeling in batch AD operation. Modeling of batch AcoD is still an effective way to interpret the BMP data quantitatively. Moreover, although being operated in different modes, both modes' fundamental laws are essentially the same. The mathematical connection between the batch and continuous models should be related in a meaningful manner or possibly unified.

AD modeling started in the early '70s. The earlier approach focused on specifying the rate-limiting steps of the multistep process [15]. Two commonly-assumed limiting steps are methanogenesis and the hydrolysis of suspended solids [16]. Later some researchers considered the concentration of volatile fatty acids as the critical parameter. Then acidogenesis and acetogenesis and the hydrogen partial pressure as key regulatory parameters were incorporated into the models separately [17]. From the early 90's up to 2002, the AD process's microbiological studies were intensified, and the IWA Task Group established the ADM1 model for Mathematical Modeling of Anaerobic Digestion Processes [18].

Even after establishing the ADM1 model and its extensive applications, its drawbacks and complexity have compelled many researchers to develop simplified alternatives. Among the simplified models, the models that consider two-reactions (acidogenesis and methanogenesis), particularly that developed by Bernard et al. [19], have been widely applied for control and optimization of AD processes and mathematical analysis [20]. For interpreting BMP assays' data, recently, Rakmak et al. [21] developed Monod-type two -substrate models for batch AD co-digestion. Later, it was used to describe the kinetics of AD co-digestion of distillery wastewater and molasses/glycerol waste in batch reactors [11].

### 1.3 Modeling of AD batch anaerobic mono and co-digestion of POME

Anaerobic co-digestion (AcoD) is a promising technique to enhance methane yield and concentration in biogas. Biogas plants from POME are relatively stable, easy to start-up and control, thus providing an excellent co-digestion platform. Different kinds of animal dung [22], rubber-latex effluent, aerobic and anaerobic sludges were used to co-digest with POME to improve C/N balance and provide richer trace elements [23].

POME's anaerobic digestion modeling is currently somewhat limited, but research in this field is expanding rapidly in recent years [23, 24, 25, 26]. Ramadhani et al. [24] studied the kinetics of anaerobic digestion of POME in batch double-stage batch anaerobic fluidized bed reactors (AFBR). The model assumed that POME substrate components could be divided into volatile fatty acid (VFA) and soluble COD. Thus, two groups of microbes are responsible for consuming them. Contois kinetics was chosen to represent the microbial growth. The model described the AD batch AFBR well, but the data were limited to verify the model. Thongpan et al. [25] used simple Monod kinetics to describe POME mono-digestion in batch and continuous AD. The simple model described the effect of the POME/sludge ratio on methane yield in batch AD very well. It also predicted the stability of continuous AD operation at different HRT correctly.

Zinatizadeh et al. [27] used Chem and Hashimoto kinetic equation and a simplified Monod model successfully to describe the kinetics of POME AD in a lab-scale up-flow anaerobic sludge fixed film (UASFF) reactor at 38 °C. They found a linear relationship between methane production rate and substrate consumption rate in the POME's COD range of 5,260–34,725 mg/l.

Using Monod kinetics with sulfate inhibition, Yingthavorn et al. [23] developed a model to describe the instability of co-digestion of POME and rubber factory effluent on an industrial scale. After calibration with data from a commercial operation, the model could reasonably explain the system's failure of the system, but the prediction was rough and required further refinement.

Other models, including ADM1, regression models, response surface methodology, fuzzy-neural net, and theoretical methane yield, were used to study POME AD digestion. However, either they are over-complicated, too empirical, or have limited mechanistic meanings, unsuitable for describing ABE or batch AcoD data for industrial applications [28, 29].

The objectives of the work are three-fold. Firstly, a multi-substrates dynamic model based on Monod kinetics and the "principle of parsimony" is developed to describe the accumulated biogas/methane curves in BMP assays and batch AcoD of POME. Secondly, we will outline the solutions of the model's equations in sufficient detail and highlight any challenge and difficulty that requires unique treatments. The answers to the problems will be suggested as possible. Thirdly, we will use the model to describe the effect of different mixing ratios between POME and chicken manure/glycerol on the co-digestion. We also conducted a limited continuous AD experiment to show the model's potential for continuous AD operations. The ultimate goals are to obtain more mechanistic understanding and pave the way for subsequent experiments (usually the continuous ones) or directly guiding for industrial applications.

## 2 Materials and methods

### 2.1 Waste and wastewater used as the substrates for co-digestion

Palm oil mill effluent (POME) samples were collected from Palmdee Sri Nakhon Company Limited, Huasai district, Nakhon Si Thammarat, Thailand. The samples were stored at the temperature of 4 °C before being analyzed and used in the co-digestion experiments.

Dry chicken manure (CM) was obtained from a community farm in Ta Sala district, Nakhon Si Thamarat. It was diluted by adding an equal mass of water, mixed together to facilitate the substrate's uniformity, and stored at room temperature (28–30 °C) before used in co-digestion.

Crude glycerol (Gly) (residue from transesterification of crude palm oil) was obtained from the biodiesel pilot project, Prince of Songkla University, Hat Yai, Thailand.

### 2.2 Analytical procedure

Wastewater analyses were carried out according to The Standard Methods for Examination of Water and Wastewater (APHA/AWWA/WEF, 2005) [30]. A gas chromatograph (Agilent Technologies Model 7890B) was used for gas analysis of biogas.

### 2.3 Biochemical methane potential (BMP) assay for co-digestion experiments

The BMP assays were conducted in batch mode, and the digesters were maintained at 28±1 °C in a temperature-controlled room. The 300-ml-volume serum bottles having a working volume of 200 ml were used as the reactor in all experiments. The tests were conducted using the method proposed by Owen et al. [31] with at least three replications. The initial pH for all reactors was adjusted to 7.0–7.5 by adding 1 N NaOH. The digesters were sealed with rubber plugs and tied up with aluminum caps. Biogas production was measured daily by the water displacement method as used by other authors [32, 33]. The methane content was measured using a Gas Chromatograph (Agilent Technologies Model 7890B). The experimental setup is summarized in Table 1.

**Table 1:** The experimental design for studying the co-digestion of POME with CM and Gly. All treatments are triplicate.

POME (ml)	Inocula (ml)	Total working volume (ml)	Label	Chicken manure (g)	Label	Crude glycerol (ml)
40	160	200	CM_0_	0	CG_0_	0
40	160	200	CM_5_	5	CG_2_	2
40	160	200	CM_10_	10	CG_4_	4
40	160	200	CM_15_	15	CG_6_	6
40	160	200	CM_20_	20	CG_8_	8
40	160	200	CM_25_	25	CG_10_	10
40	160	200	CM_30_	30		-
40	160	200	CM_35_	35		-
40	160	200	CM_40_	40		-
40	160	200	CM_45_	45		-
40	160	200	CM_50_	50		-

Biochemical methane potential (BMP) was estimated from the accumulated biogas at the end of the assay and the average methane content.

### 2.4 Anaerobic digestion of POME in a completely stirred tank reactor (CSTR)

A limited CSTR experiment was carried out to partially validate the applicability of process parameters obtained from the BMP tests. The 14-Liter CSTR reactor was started up by filling with 50% POME (74,240 mgCOD/l) and 50% active AD sludge obtained from a biogas plant in Nakhon Si Thammarat province. The start-up period was 14 days. The reactor was then switched to a semi-continuous mode at 30-days HRT by feeding the reactor twice a day and prolonged this mode for three months to test for the process stability. However, the data were collected and reported up to 45 days. Only accumulated biogas and COD of digestate are reported in this article.

## 3 Model development and parameter estimation

In this work, our modeling targets are to develop tools for describing the kinetics of co-digestion of POME and CM/Gly and an attempt to bridge to gap between BMP assays (or batch AD co-digestion) and direct industrial applications. Thus the model should meet the following requirements:

1. Its structure should be based on fundamental, well-established kinetics such as Monod-type kinetics
2. The model should be flexible and extensible, providing building blocks for BMP assay, batch, continuous AcoD, and phenomenal transport models from lab-scale to industrial reactors. However, this article targets BMP tests and batch AcoD with a minimal set of measured data: accumulated/daily biogas, COD, or VS. Other variables are optional, albeit providing more in-depth information about the system's states.
3. Based on the principle of parsimony, the model should contain only measured or observable variables, with the associated identifiable parameters. Parameter identifiability means that all parameters included in the model structure should pass a significant test at an acceptable confidence level or have fundamental meaning inseparable from the model.
4. It should be in-line with the well-established AD models such as ADM1 [18], AMOCO [19], but emphasizing substrate categories that fit intuitively with the typical accumulated biogas/biomethane (ABE) curves' characteristics of batch AcoD experiments.

### 3.1 Typical accumulated biogas/methane curves (AB curves) observed in the co-digestion experiments

**Figure 1:**
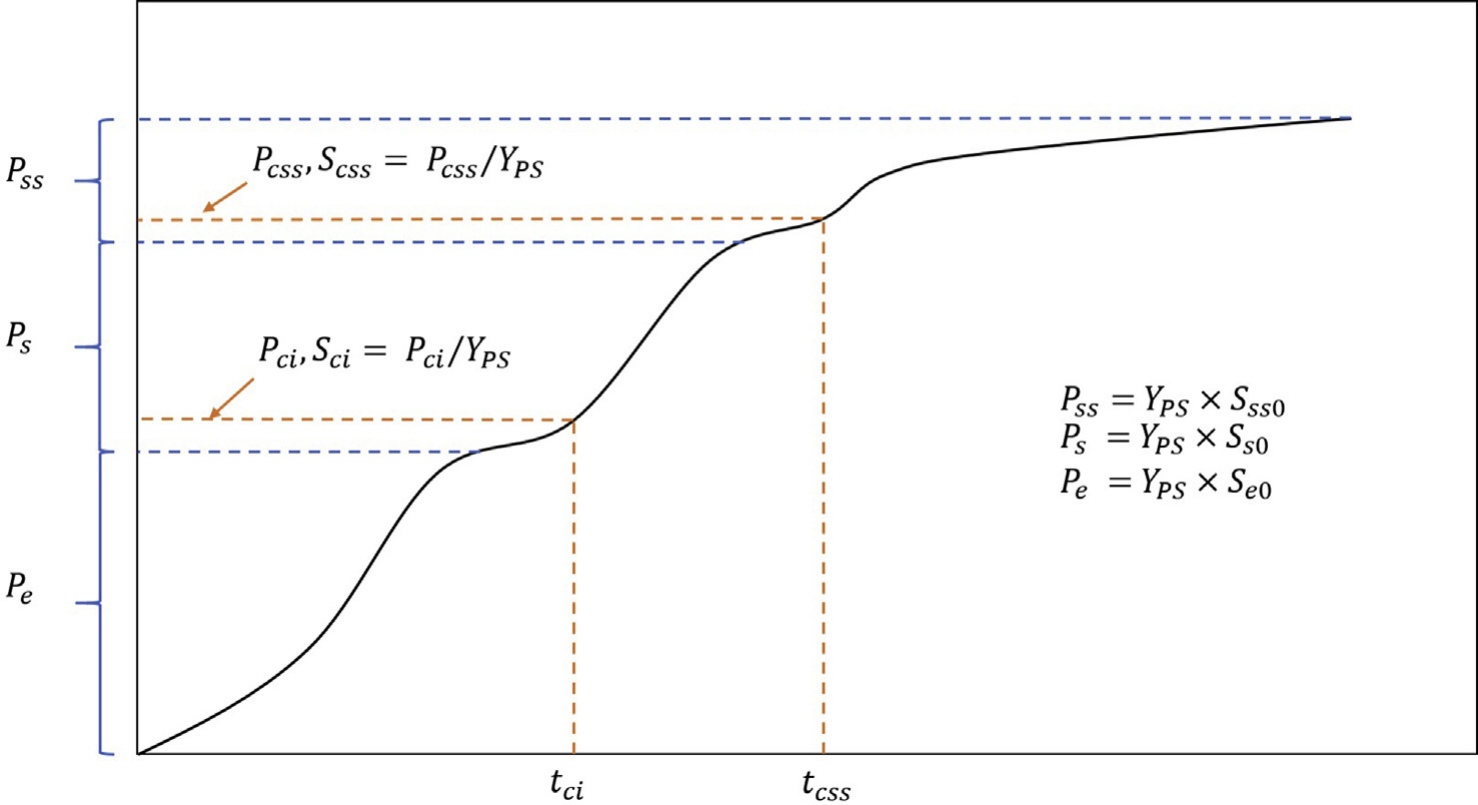
A typically accumulated biogas (or ABE) curve frequently occurring in the co-digestion of POME and chicken manure or POME with glycerol.

The experimental data obtained from batch co-digestion of POME or BMP assay with various co-substrates including chicken manure and glycerol, the following ABE curves pattern (Figure 1) recurs very often and consistently. The pattern implies that there are multiple kinds of substrates being digested serially or in parallel. This observation agrees with the substrates classification in the ADM1 model: easily degradable, slowly degradable substrates. However, the classification (as in the ADM1 model) does not consider the microbial substrate preferences but allows parallel and serial reactions. From our experiences in AD modeling using similar classified substrates as in ADM1, the models could not replicate the trends of the curves unless it can characterize the preferential transitions between different groups of substrates.

### 3.2 The proposed multi-substrate monod kinetics

**Figure 2:**
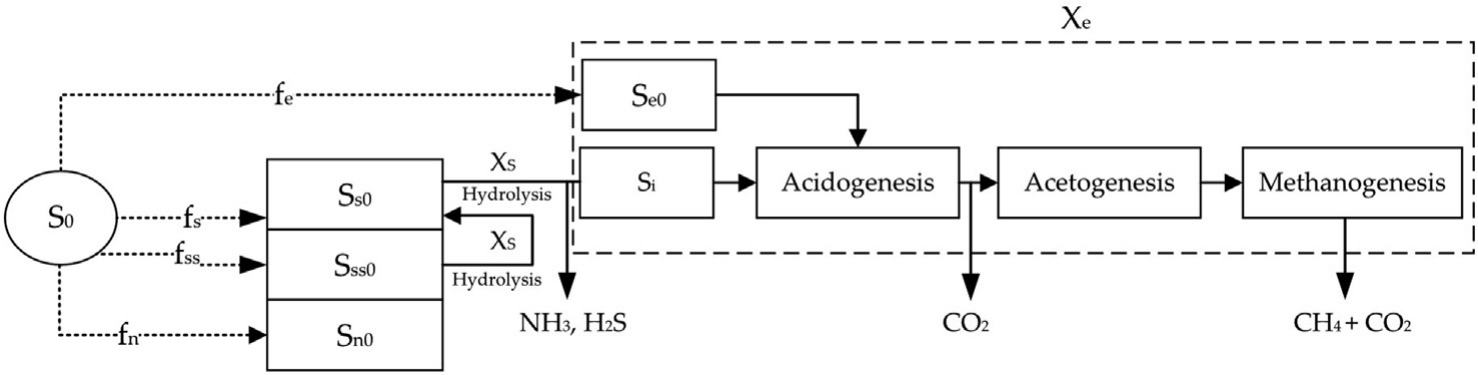
A conceptual conversion diagram showing the substrate categories and the sequences of substrate conversion.

The observed ABE curves suggest multiple COD (substrate) groups in the wastewater with different degradability levels and microbial preferences. Referring to Figure 1, we classify the wastewater COD into three categories: easily (ED), slowly (SD), and very slowly degradable (VSD). The acidogenic and acetogenic bacteria can readily consume the substrates in the first category. On the contrary, substrates in the second category require hydrolysis, and it becomes ED before further consumption by acidogenic bacteria. Note that the ED substrates produced from SD may differ from the existing ED substrates regarding composition and microbial preference. VSD are those big particles difficultly degradable in a typical mesophilic environment. Thus, they are degraded very slowly in the digestion timeframe. The diagram in Figure 2 visualizes the structure of the model.

In Figure 2, $S_{0}$ is the total initial substrate concentration (mgCOD). $S_{e0},S_{s0},S_{ss0}$ and $S_{n0}$ are the initial concentration of easily, slowly, very slowly degradable, and non-degradable substrates, respectively. $f_{e},f_{s},f_{ss}$ and $f_{n}\;$are the easily, slowly, very slowly, and non-degradable fractions of the influent. We assume two groups of microbe working sequentially: hydrolytic bacteria ($X_{s}$) and biogas producing bacteria ($X_{e}$). The hydrolytic bacteria ($X_{s}$) is responsible for converting complex and large molecules into small molecules and, finally, easily degradable substrates.

Note that, essentially, the model becomes a two-agent and four-substrate model. Mathematically, based on Monod kinetics, we have the following system of algebraic-differential equations.

For microbial growth, the Monod kinetics is used for all types of substrate consumption.

(1) $$\mu_{e}=\;\frac{\mu_{me}\;S_{e}}{K_{Se}+S_{e}},\;\mu_{i}=g_{i}\;\frac{\mu_{mi}\;S_{i}}{K_{Si}+S_{i}},\;\mu_{s}=\;\frac{\mu_{ms}\;S_{s}}{K_{Ss}+S_{s}},\;\mu_{ss}=\;g_{ss}\frac{\mu_{mss}\;S_{ss}}{K_{Sss}+S_{ss}}$$

Where, $\mu_{e},\;\mu_{i},\;\mu_{s},\;\mu_{ss}$ are the specific growth rate of the microbes consuming easily, intermediate (the products of the degradation of SD substrates), slowly, and very slowly degradable substrates, respectively. Similarly, $S_{Se},S_{Si},\;S_{Ss},\;S_{Sss}$, $\mu_{me},\;\mu_{mi},\;\mu_{ms},\;\mu_{mss}$ and $K_{Se},\;K_{Si},\;K_{Ss},\;K_{Sss}$ are the concentration (mg/l), maximum specific growth rates (d^−1^), and half-saturation constants (mg/l) for the corresponding substrates.

$g_{i}$ and $g_{ss}$ are the preference or switching function that characterizes particular groups of microbes over different consumable substrates. For example, biogas producing bacteria (acidogens, acetogens, and methanogens) may first consume the ED substrates. If ED substrates are exhausted to a threshold level, they start consuming the intermediate ones. The microbial consuming behavior is represented by "*a preference function or a switching function*." There are many candidate functions for expressing a microbial preference, such as logistic and arctan function. In this work, we chose the arctan function of the following forms.

(2) $$g_{i}=\;\frac{1}{\pi }\left(tan^{-1}\left(\kappa_{i}Y_{PS}\left(S-S_{ci}\right)\right)+\frac{\pi }{2}\right)\;$$

(3) $$g_{ss}=\;\frac{1}{\pi }\left(tan^{-1}\left(\kappa_{ss}Y_{PS}\left(S-S_{css}\right)\right)+\frac{\pi }{2}\right)\;$$

Here $g_{i},g_{ss},\kappa_{i},\kappa_{ss},S_{ci},S_{css}\;$are the preference functions and their corresponding parameters. $Y_{\mathrm{PS}}$ is the substrate-biogas yield coefficient (ml/mgCOD), assuming constant regardless of the substrate type. S is the total substrate, the sum of all substrate categories ($S_{e},S_{i},S_{s},\;S_{ss}S_{n}$).

**Figure 3:**
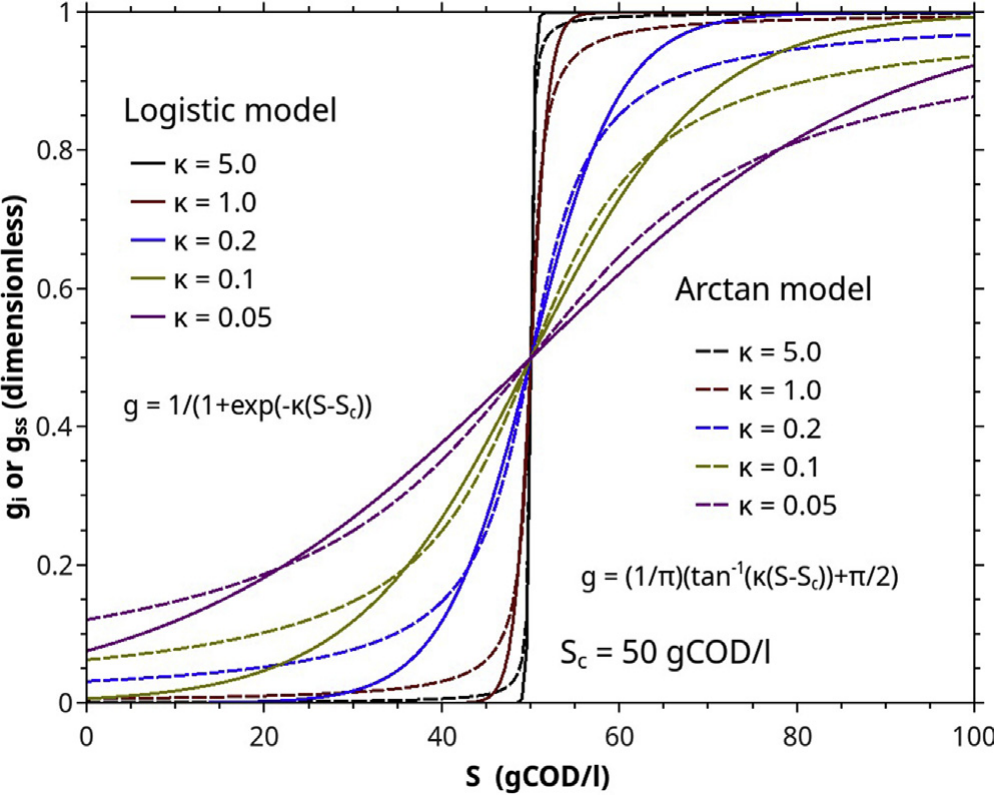
The behavior of two preference/switching functions: logistic and arctan functions. ($S_{n}$= 50 gCOD/l).

The functions act as a switch, turning the factor ($g_{i},\;g_{ss}$) of the associated specific growth rates from zero to one. $S_{c}$ specifies the deflection point where the functions equal to 0.5, and κ describes the transition behavior (Figure 3).

Therefore, the following differential equations represent cell growth rates.

(4) $$\frac{dX_{e}}{dt}=\;\left(\mu_{e}+\mu_{i}-k_{de}\right)X_{e}$$

(5) $$\frac{dX_{s}}{dt}=\;\left(\mu_{s}-k_{ds}\right)\;X_{s}$$

Where $X_{e},X_{s}$ and $K_{de},K_{ds}$ are microbial activity and the decaying rate of the corresponding microbes.

The following differential equations describe the degrading rate of each substrate.

(6) $$\frac{dS_{e}}{dt}=\;-\frac{\mu_{e}}{Y_{XeSe}}\;X_{e}$$

(7) $$\frac{dS_{i}}{dt}=f_{is}\frac{1-Y_{XsSs}}{Y_{XsSs}}\;\mu_{s}X_{s}-\frac{\mu_{i}}{Y_{XeSi}}\;X_{e}$$

(8) $$\frac{dS_{ss}}{dt}\;=\;-\frac{\mu_{s}}{Y_{XsSs}}X_{s}+f_{SsX}\left(k_{ds}\;X_{s}+k_{de}X_{e}\right)+\frac{\mu_{ss}}{Y_{XsSss}}X_{s}$$

(9) $$\frac{dS_{ss}}{dt}=\;-\;\frac{\mu_{ss}}{Y_{XsSss}}\;X_{s}\text{, and }\frac{dS_{n}}{dt}=0$$

(10) $$\frac{dS}{dt}\;=\;\frac{dS_{e}}{dt}+\frac{dS_{s}}{dt}+\frac{dS_{ss}}{dt}+\frac{dS_{n}}{dt}$$

And the product formation is described by the following equations.

(11) $$\frac{dP_{e}}{dt}=\frac{\mu_{e}Y_{PSe}}{Y_{XeSe}}\;X_{e}$$

(12) $$\frac{dP_{i}}{dt}=\frac{\mu_{i}Y_{PSi}}{Y_{XeSi}}\;X_{e}$$

(13) $$\frac{dP}{dt}=\frac{dP_{e}}{dt}+\frac{dP_{i}}{dt}$$

$f_{is}$ are the fraction of slowly degradable substrate converted to the intermediate substrate.

With proper initial conditions, this system of equations can be solved using standard numerical methods.

### 3.3 Multi-substrate monod model for CSTR

The multi-substrate Monod model can be easily modified for CSTR by adding the influent and effluent terms, ignoring the nutrient preferences, and the following system of ODEs is obtained.

(14) $$\frac{dX_{e}}{dt}=\left(\mu_{e}+\mu_{i}-k_{de}\right)X_{e}+\alpha \frac{q_{in}}{V_{L}}\left(X_{e,in}-X_{e}\right)$$

(15) $$\frac{dX_{s}}{dt}=\left(\mu_{s}-k_{ds}\right)X_{s}+\alpha \frac{q_{in}}{V_{L}}\left(X_{e,in}-X_{e}\right)$$

(16) $$\frac{dS_{e}}{dt}=-\frac{\mu_{e}X_{e}}{Y_{XeSe}}+\alpha \frac{q_{in}}{V_{L}}\left(S_{e,in}-S_{e}\right)$$

(17) $$\frac{dS_{i}}{dt}=f_{is}\frac{1-Y_{XsSs}}{Y_{XsSs}}\mu_{s}X_{s}-\frac{\mu_{i}}{Y_{XeSi}}X_{e}+\frac{q_{in}}{V_{L}}\left(S_{i,in}-S_{i}\right)$$

(18) $$\frac{dS_{s}}{dt}=-\frac{\mu_{s}}{Y_{XsSs}}X_{s}+f_{SsX}\left(k_{ds}X_{s}+k_{de}X_{e}\right)+\frac{\mu_{ss}}{Y_{XsSss}}X_{s}+\frac{q_{in}}{V_{L}}\left(S_{s,in}-S_{s}\right)$$

(19) $$\frac{dS_{ss}}{dt}=-\frac{\mu_{ss}}{Y_{XsSss}}X_{s}+\frac{q_{in}}{V_{L}}\left(S_{ss,in}-S_{ss}\right)$$

(20) $$\frac{dP}{dt}=\left(Y_{PSe}\frac{\mu_{e}}{Y_{XeSe}}+Y_{PSi}\frac{\mu_{i}}{Y_{XeSi}}\right)X_{e}$$

where subscript "in" indicates the components of influent wastewater. α is the fraction of cells removed by the dilution effect. α=1 is for a completely mixed reactor, while α=0 means that all biomass remains in the reactor. $q_{in}$ and $V_{L}$ are the influent flow rate and liquid volume of the reactor, respectively.

### 3.4 Model fitting and parameter estimation

The multi-substrate model has twenty-one parameters ($\mu_{me},\;\mu_{mi},\;\mu_{ms},\;\mu_{mss},K_{Se},K_{Si},K_{Ss},$ $K_{Sss,}Y_{XeSe},Y_{XsSs},Y_{XsSss},Y_{PSe},\;Y_{PSi},f_{is},f_{Ssx},\;\kappa_{i},\kappa_{ss},\;k_{de},k_{ds},S_{ci},S_{css}$) and six initial conditions $\left(X_{e0},\;X_{s0},S_{e0},S_{i0},S_{s0},S_{ss0},S_{n0}\right)$. At the initial time $P\;=\;P_{e\;}=P_{i}=0$. We assume that only a minimum data are available in fitting the model to experimental data, including accumulated biogas/methane (ABE) data, initial/final COD (or VS). Any other data about initial conditions are optional because they are generally observable and can be estimated uniquely (with some confidence levels) using model simulation, iterative search, and curve-fitting.

To simplify further the parameter estimation, we assume that all half-saturation constants are equal. That is $K_{S}=K_{Se}=K_{Si}=K_{Ss}=K_{Sss},\;$ albeit must be estimated by curve-fitting. Similarly, follow Rittman and McCarty [34], we assume that all cell yield coefficients are equal and 1 g of degradable COD only produces 0.1 g biomass COD, $Y_{XS}=\;Y_{XeSe}=Y_{XeSi}=Y_{XsSs}=Y_{XsSss}=\;0.1$. Other fixed parameters are the microbial death rate ($k_{d}=\;k_{de}=\;k_{ds}$ = 0.05 d^−1^), is a typical values which is in the range of 0.02–0.07 d^−1^ [35, 36, 37]. The preference parameters ($\kappa_{i}\;=\;\kappa_{ss}=\;0.1$) suggest a moderately smooth transition (see Figure 3). The fractions $f_{is},\;f_{SsX}$ are 1.0 and 0.7 respectively. That is we assume that all SD substrates end up as intermediate/easily degradable ones. $f_{SsX}$ has a very minor effect on the overall ABE curves because of the low microbial death rate.

Finally, seven parameters remain to be estimated: $\mu_{me},\;\mu_{mi},\mu_{ms},\mu_{mss},K_{Se}$, $S_{ci},S_{css}$ and $Y_{ps}$. From our preliminary sensitivity analysis, these seven parameters are among the most sensitive and meaningful in characterizing the underlining kinetics of batch co-digestion. Among the initial conditions ($X_{e0},\;X_{s0},S_{e0},S_{i0},S_{s0},S_{ss0},S_{n0})$ typical BMP assays only provide the total COD or VS, $S\;=\;S_{e0}+S_{i0}+S_{s0}+S_{ss0}+S_{n0}$. However, we can estimate $S_{n0}$ from the ABE curves by extrapolating the curves to infinite time. If substrate consumption is somewhat sequential, we can recognize $S_{e0},S_{s0},S_{ss0}$visually, and estimate them by graphical methods. Nevertheless, the final best estimates of $\;S_{e0},S_{s0},S_{ss0}\;$were obtained from coupling the non-linear curve-fitting routines with the trial-and-error technique. We use the adjusted coefficient of determination (adjusted R^2^) as the criteria to terminate the iterations. At t=0, $S_{i0}\;=\;0$ because it is considered as part of $S_{e0}$. The parameters $X_{e0}$ can be estimated from the initial part of ABE curves. We assume that $X_{s0}$ is proportional to $X_{e0},\;\left(S_{s0}+S_{ss0}\right)/S_{e0}$ and $X_{s0}\;=X_{e0}\;\left(S_{s0}+S_{ss0}\right)/S_{e0}.$

**Figure 4:**
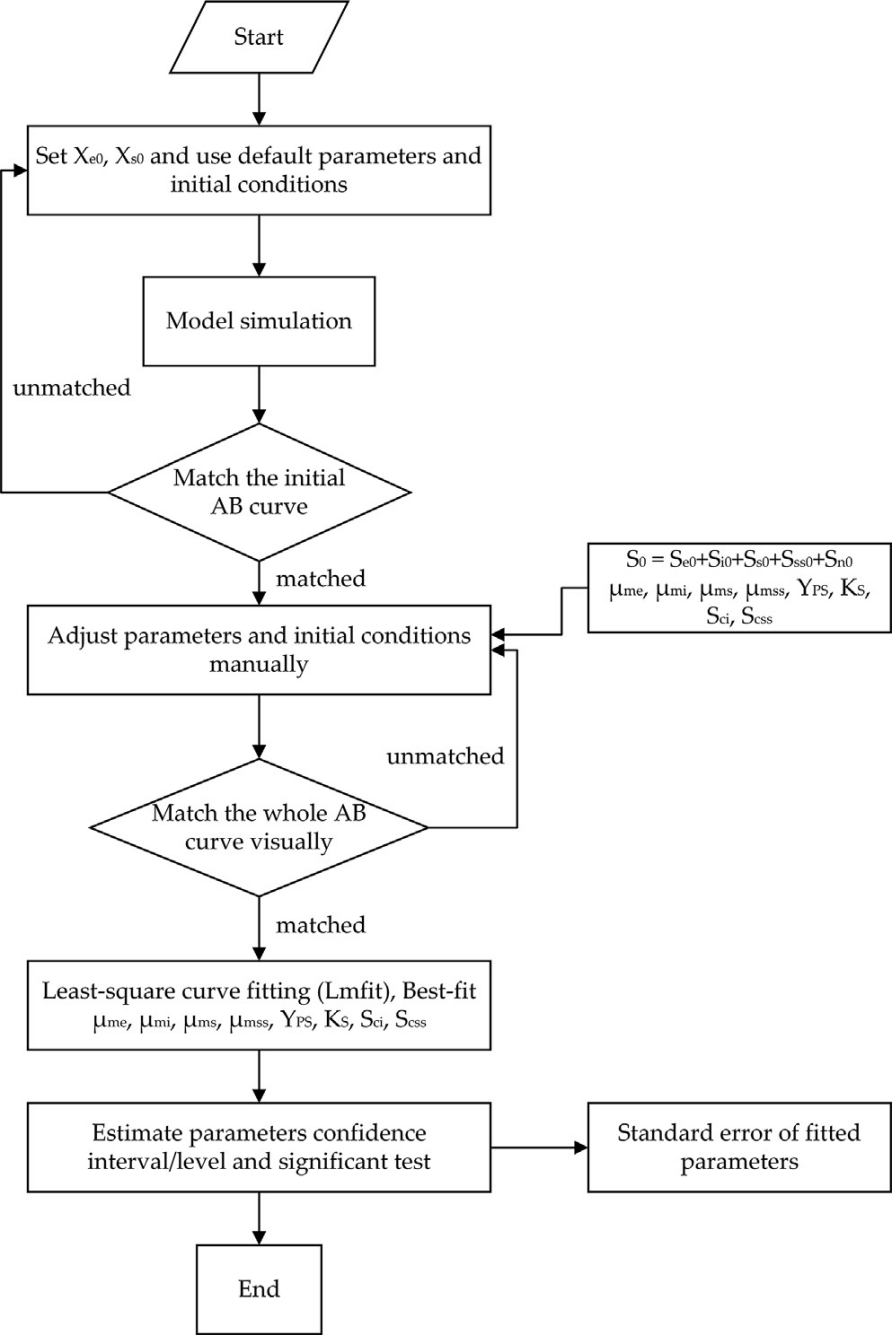
Model fitting strategy for the multi-substrate model, parameters' confidence interval, and significance.

From the authors' experiences, multi-substrate model fitting requires a few preliminary steps before sending the initial guesses to a non-linear curve-fitting software to converge to the final well-fit model for an ABE data set. The fitting process was as follows and depicted in Figure 4.

1) A set of default or assumed parameters ($\mu_{me},\;\mu_{mi},\mu_{ms},\mu_{mss},K_{Se}$, $S_{ci},S_{css}$ and $Y_{ps}$) were prepared, and a complete set of initial conditions was specified. For convenience, $P_{ci},\;P_{css}$ are specified instead of $S_{ci},S_{css}$. They are related according to the following mass balances.

$$S_{ci\;}=\;S_{0}-P_{ci}/Y_{PSe}\text{ and }S_{css\;}=\;S_{0}-P_{css}/Y_{PSe}$$

2) The model simulation started for visual parameter adjustment. Then we adjusted the parameters iteratively to find a candidate model by visual judgment.
3) Iteratively (by intuitive trial-and-error) set parameters and the initial conditions until the predicted curves and data are close together.
4) Then, we start model-fitting using a non-linear optimization routine (LMFIT) to obtain the best-fit parameters. If the results are satisfactory, then the process is stopped. Otherwise, the procedures have to repeat iteratively.

### 3.5 Statistical analysis

LMFIT [38], the non-linear least-squares minimization and curve-fitting for Python language, was used for model fitting and parameter estimation. Parameters were tested for significance (95% confidence level or α=0.05) using Student's t-distribution with n−p degrees of freedom, where *n* is the number of measurements, and p is the number of parameters. The 1−α confidence interval for the parameter $b_{j}$ in the model can be calculated as follows:

(21) $$b_{i}\pm se\left(b_{i}\right)t\left(n-p;\alpha /2\right)$$

Where t(n−p;α/2) is the Student's t-distribution with an n-p degree of freedom. In other words, the parameter is significant at a 95% confidence level if $b_{i}/se\left(b_{i}\right)>t\left(n-p;\alpha /2\right)$ ≅2.00. The adjusted R^2^ is defined as follows.

(22) $$R^{2\;}=1-\;\frac{SS_{residuals}\;}{SS_{total}},\;and\;R_{adj}^{2}=1-\;\frac{SS_{residuals}/\left(n-p\right)\;}{SS_{total}/\left(n-1\right)}$$

Where $SS_{residuals}$ is the sum-of-square of the discrepancy between the model prediction and the data. $SS_{total}$ is the total sum-of-squares of the difference between the overall mean of the dependent variable and the data. The overall model significance test is based on the F-distribution.

(23) $$F\left(p-1,n-p\right)\;=\;\frac{R^{2}/\left(p-1\right)}{\left(1-R^{2}\right)/\left(n-p\right)}$$

For our model and ABE data, p-1 = 6, and n-p > 40. Thus the model is statistically significant if F > 1.92. It can be shown that all our model-data fitting resulted in F >> 1.92. Therefore, there is no need to report the results of significance tests for the overall model. Therefore, we only present the standard error of estimation for each parameter.

## 4 Results and discussions

### 4.1 Basic chemical properties of the substrate

The basic chemistry of POME, chicken manure, and crude glycerol are summarized in Table 2. The values of the chemical parameters of POME and CM are in the typical range [39]. The COD of crude glycerol is similar to those given by [40, 41].

**Table 2:** Basic chemical properties of POME, chicken manure, and glycerol used in this work.

Parameter	POME	CM (diluted with water 1:1)	Crude glycerol
pH	4.72 ± 0.05	8.4 ± 0.8	9.5 ± 0.7
COD (g/L)	78.83 ± 9.33	102.55 ± 12.45	1065 ± 165
TS (g/L)	53.09 ± 3.53	78.68 ± 7.57	NA
VS (g/L)	44.26 ± 1.16	56.03 ± 5.98	727.75 ± 27.75
Alkalinity (g CaCO_3_/L)	4.76 ± 0.14	5.5 ± 0.2	NA
VFA (g/L)	6.79 ± 0.50	1.23 ± 0.13	NA
C/N ratio	21.74 ± 1.11	9.28 ± 0.82	717.4 ± 95.1
Carbon (%w/w)	2.05 ± 0.20	9.21 ± 0.15	34.53 ± 4.00

Remark: The table's values are the average values ± the lower and upper limits of the measured values.

Note that POME is mildly acidic. CM is slightly alkali, where crude glycerol can be highly alkali because of alkali (i.e., KOH, NaOH) residues. Higher total solid (TS) in CM than POME indicates a higher fraction of non-degradable substrate present in CM. Many AD researchers suggested that the optimal range of C/N ratio is 20–30 [42,43]. Thus POME has an optimal C/N ratio, where CM is a rich N with a low C/N ratio. Gly alone is not suitable for microbial growth because of its very high COD, high pH, and imbalance C/N ratio. However, very high COD makes Gly very ideal for being additive to enhance biogas production.

### 4.2 Co-digestion of POME with chicken manure: BMP and %COD removal

Table 3 summarizes the BMP assays' main parameters for the co-digestion between POME (40 ml) and different amounts of CM (0–50 mg).

**Table 3:** Main parameters of the BMP experiments: co-digestion of POME and CM.

Digester	pH		Alkalinity (mg/L asCaCO_3_)		VFA (mg/L asCH_3_COOH)		VFA/ALK		COD (mg/l)		%COD removal
	0 d	55 d	0 d	55 d	0 d	55 d	0 d	55 d	0 d	55 d	55 d
CM_0_	7.8	7.9	1,812	2,434	144	109	0.10	0.05	80,640	15,700	80.5
CM_5_	7.87	7.9	1,990	3,470	261	245	0.13	0.07	87,760	15,920	81.9
CM_10_	7.8	7.93	3,735	3,154	388	96	0.10	0.03	92,800	17,800	81.8
CM_15_	7.6	7.83	3,012	3,573	343	236	0.11	0.07	89,920	17,050	80.8
CM_20_	7.63	7.93	3,316	4,080	949	423	0.30	0.10	85,440	16,540	80.6
CM_25_	7.73	7.87	4,207	3,115	1,482	405	0.35	0.13	85,200	18,740	78.0
CM_30_	7.63	7.87	4,356	3,662	2,719	475	0.62	0.13	89,600	17,480	80.5
CM_35_	7.57	5.17	4,563	2,856	2,686	332	0.59	0.12	86,240	24,660	71.4
CM_40_	7.7	8.1	4,071	4,974	2,332	772	0.57	0.16	91,160	26,190	71.5
CM_45_	7.5	5.43	3,853	2,998	1,289	492	0.34	0.16	93,120	22,490	75.8
CM_50_	7.63	7.97	5,396	5,599	1,630	1164	0.30	0.21	92,480	24,310	73.7

**Figure 5:**
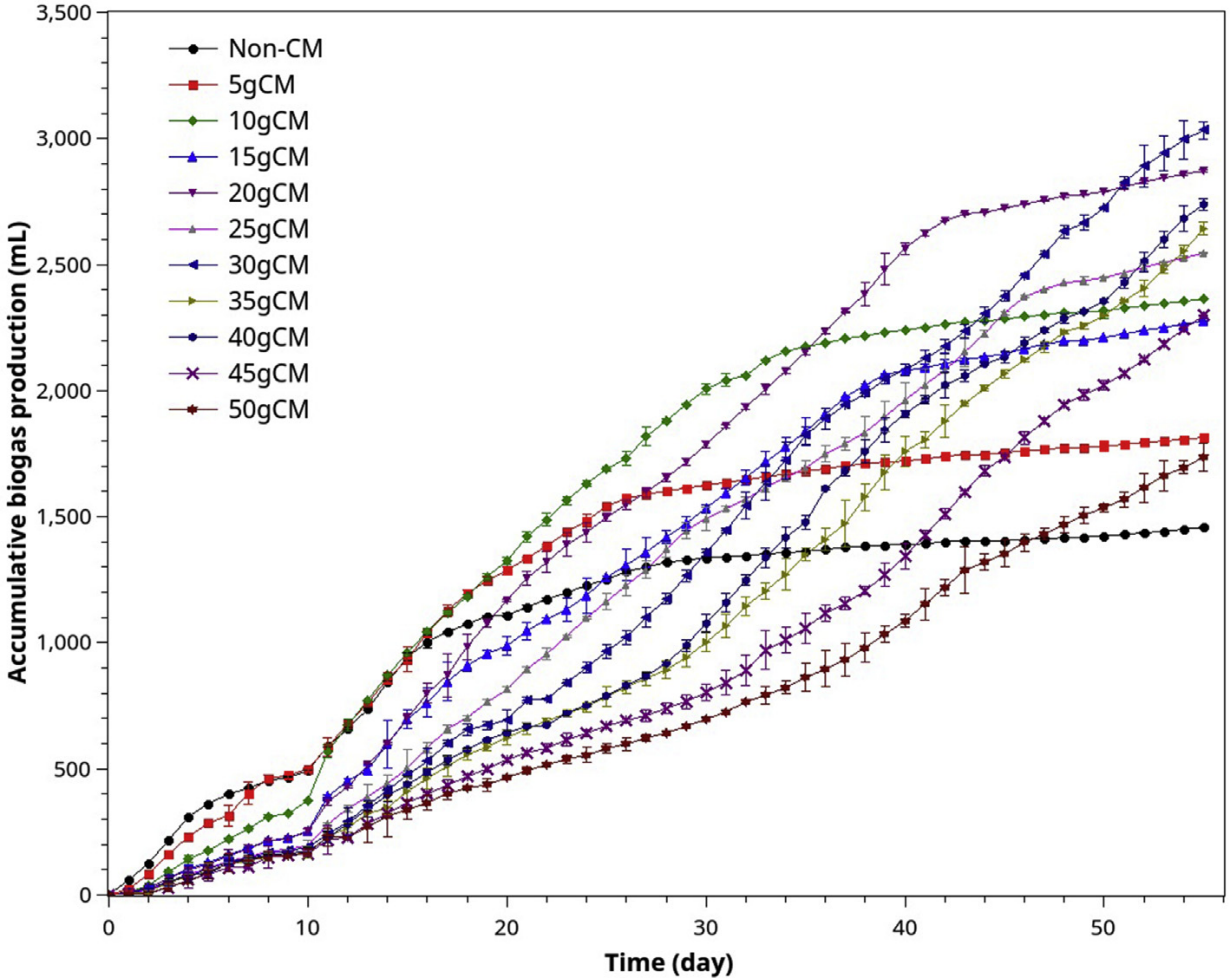
Accumulated biogas evolution (ABE) curves resulting from co-digestion of POME and different amounts of chicken manure.

In Table 3, we notice that the pH was stable within the optimal range (pH 7.1–7.8) [44,45] through the digestion process unless CM excessed 35g (POME: CM 40:35). For stable anaerobic digestion, VFA/ALK should be between 0.05-0.4. Therefore, the digestion was regular throughout the batch processes for all mixing ratios. Figure 5 shows the accumulated biogas evolution for different mixing ratios of POME and CM.

**Figure 6:**
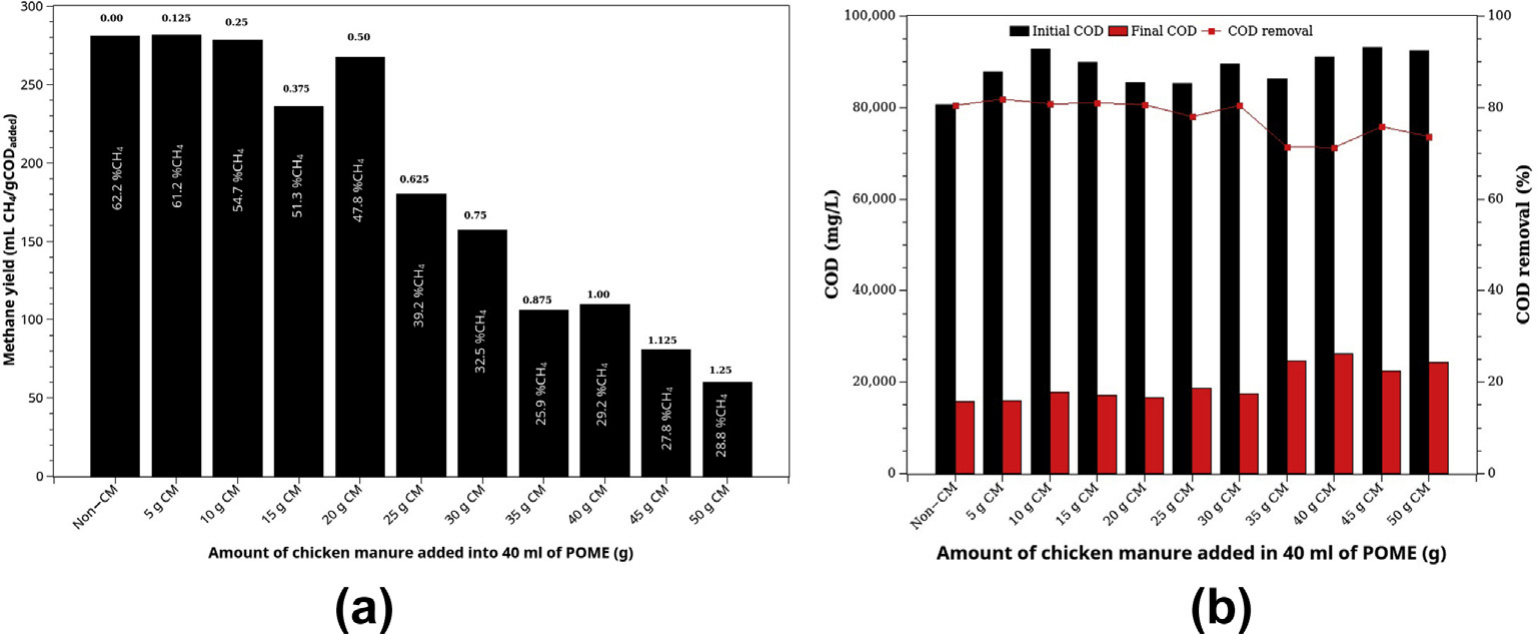
Co-digestion of POME and chicken manure: (a) methane yield (BMP) and (b) %COD removal. The numbers on top of each bar in (a) are the CM: POME ratio (g CM: mlPOME).

Figure 6a depicts the methane yields or the biochemical methane potentials. The initial, final, and %COD removal is shown in Figure 6b. In general, %COD removal was about 80% for CM: POME ratio was lower than 3/4 (30 gCM:40 ml POME). The %COD removal fell off to about 70% at the ratio of 5/4 (50 g CM:40 ml POME). However, methane yield was more sensitive to the increase of CM portion even at 2/4 (20 g CM:40 ml POME), where methane yield started to drop gradually because of the lower methane content in the biogas. However, the %COD removal dropped only slightly from 80% to around 70%, suggesting that anaerobic fermentation rather than AD dominated the degrading process. Moreover (Figure 6a), the CH_4_ in biogas reduced from 62.2% to 28.8% as the CM portion increase from 0 to 50g/40 ml POME.

### 4.3 Co-digestion of POME with glycerol: BMP and %COD removal

Table 4 summarizes the BMP assays' main parameters for the co-digestion between POME (40 ml) and different amounts of Gly (0–10 mg).

**Table 4:** Main parameters of the BMP experiments: co-digestion of POME and Gly.

Digester	pH		Alkalinity (mg/L asCaCO_3_)		VFA (mg/L asCH_3_COOH)		VFA/ALK		COD (mg/l)		%COD removal
	0 d	51 d	0 d	51 d	0 d	51 d	0 d	51 d	0 d	51 d	
CG_0_	7.0	7.37	6,275	4,757	975	430	0.1553	0.0906	82000	19,560	76.1
CG_2_	7.1	7.26	5,605	4,613	1,065	385	0.1900	0.0833	152,400	62,900	58.7
CG_4_	7.0	6.19	7,510	7,130	1,433	1110	0.1907	0.1556	232,800	115,700	50.3
CG_6_	7.0	5.58	7,940	8,570	1,470	1024	0.1851	0.1194	313,100	158,800	49.3
CG_8_	7.0	5.39	8,870	9,380	1,680	1157.5	0.1894	0.1232	393,500	183,300	53.4
CG_10_	7.0	5.15	8,915	11,430	1,538	1837.5	0.1724	0.1594	473,900	252,700	46.7

Crude glycerol (Gly) has a very dense COD. Even at a low mixing ratio of 2 ml Gly: 40 ml POME, the COD of the mixed wastewater was as high as 152 gCOD/l, which, when converted into VFA, will strongly affect the pH and inhibit microbial growth. It is evident that the Gly: POME mixing ratio higher than 1/20 pushed the AD to an unstable state. Although the VFA/ALK did not fall into an unstable range, the pH dropped out of the normal range (6.8–8.0) at a higher mixing ratio. Since the low pH was not suitable for methane-producing bacteria, %CH_4_ reduced shapely as the mixing ratio exceeded 1/20.

**Figure 7:**
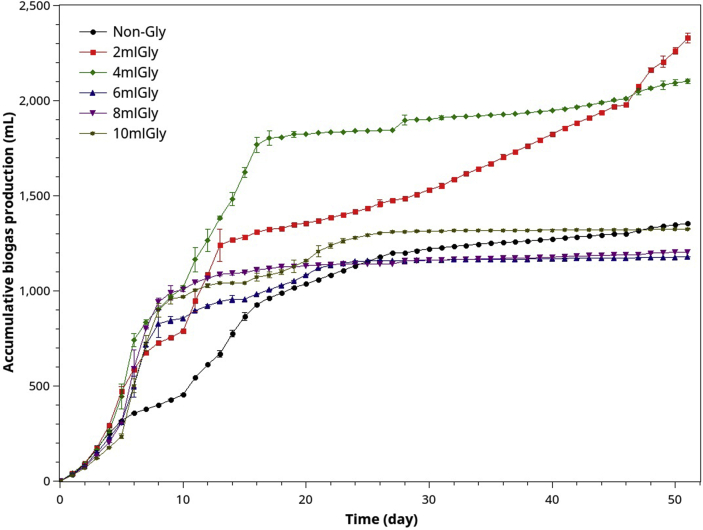
Accumulated biogas evolution (ABE) curves resulting from POME co-digestion and different amounts of crude glycerol.

**Figure 8:**
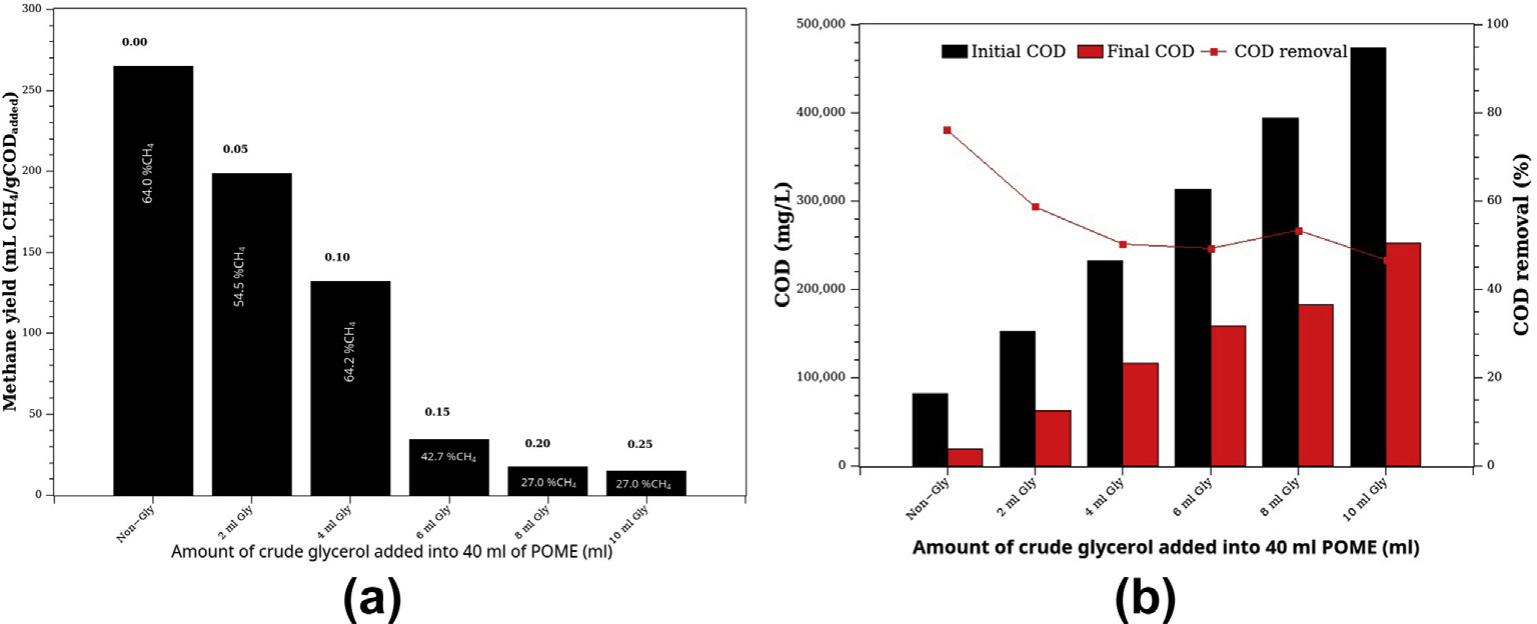
Co-digestion of POME and crude glycerol: (a) methane yield (BMP) and (b) %COD removal. The numbers on top of each bar in (a) are the Gly: POME ratio (ml Gly: ml POME). The CH_4_ concentrations of the biogas are given on the bars in (a).

Figure 7 depicts the effect of POME-Gly co-digestion at different mixing ratios on the accumulated biogas curves. Figures 8a and 8b show the methane yields at different Gly: POME mixing ratios and their effect on the %COD removal, respectively. By comparison, Gly has very high carbon content, thus high VS and COD. Adding Gly only a small amount (e.g., 2 mlGly/40 mlPOME) doubled the amount of biogas. Therefore Gly can be an excellent additive for controlling the biogas output for on-demand energy generation.

### 4.4 Fitting (calibrating) multi-substrate monod model to BMP data: POME-CM co-digestion

The original ABE of this AcoD data set is presented in Figure 5. Visual comparison can be misleading because of the varying initial COD. However, after they are normalized to one gram of initial COD, curves have consistent trends. By visual inspection, the yield did not drop significantly until the CM/PIOME ratio exceeded 25% (10 g CM: 40 ml POME). As the yield remained around 270–285 ml CH_4_/g COD in the range of 0–25% mixing ratio, we can conclude that AcoD between POME and CM did not show a significant synergistic effect. Instead, high ammonia nitrogen in CM showed an inhibitory effect at all levels (even at a 12.5% mixing ratio). However, the final yield was not affected significantly at the ratio range of 0–20%. Further increase in CM addition made the methane yield drop sharply and consistently.

The results are not surprising because POME is rich in nutrients, and its C/N ratio falls within the optimal range. Adding CM may not positively change the nutrient balance, but it allows us to obtain more biogas and provide more degrees of freedom and flexibility in waste management. From the perspective of CM as waste management, CM's co-digestion with POME solves the instability of CM single digestion, increases CM's digestibility, optimizes the methane yield (by adjusting C/N ratio and reduces the accumulation of toxic products), controlling the methane output to match with the demanding load [7, 46, 47].

It takes great care to fit the model to ABE data. Firstly, visual inspection helps identify the number of substrate groups involved and when their final conversion to methane occurs. The effect of degradability and microbial preferences was observed explicitly or hidden for the current co-digestion but mathematically observable.

**Figure 9:**
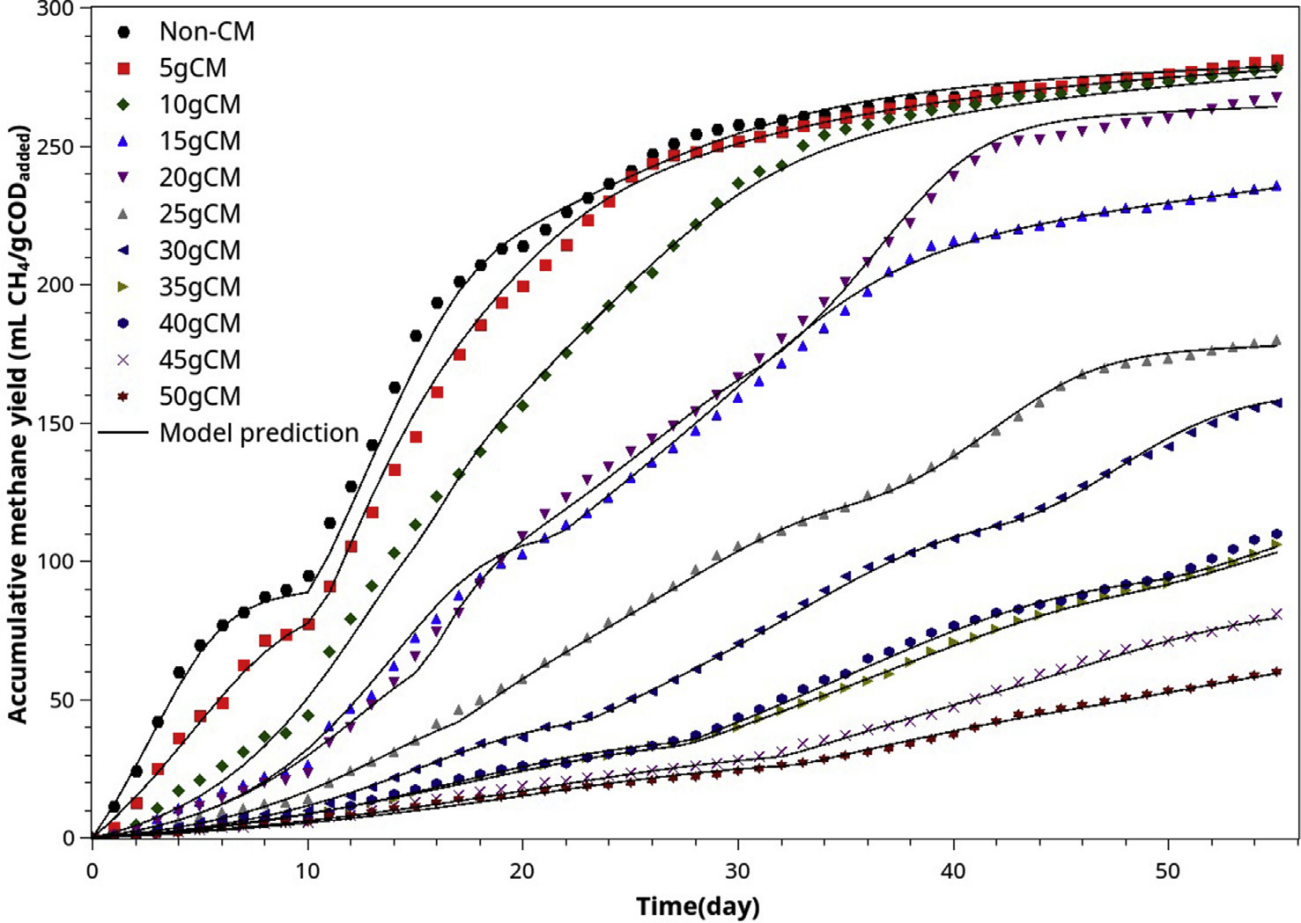
The comparison between experimental ABE curves (normalized) and the model's best-fit for the batch AcoD of POME with different CM supplement levels.

After visual inspection and manual trial-and-error fitting, we obtained sets of initial conditions and parameter estimates. They were used as inputs of LMFIT optimization and, after a few iterations, the best estimates of $\mu_{me},\;\mu_{mi},\mu_{ms},\mu_{mss},K_{Se}$, $S_{ci},S_{css}$ and $Y_{ps}$ were obtained. The results are shown in Figure 9 and Table 5. Note that $\mu_{mi}=\;f_{\mu_{mi}}\mu_{me}$, $\mu_{ms}=\;f_{\mu_{ms}}\mu_{me}$, $\mu_{mss}=\;f_{\mu_{mss}}\mu_{me}$. There are a few important observations that could be noted.

**Figure 10:**
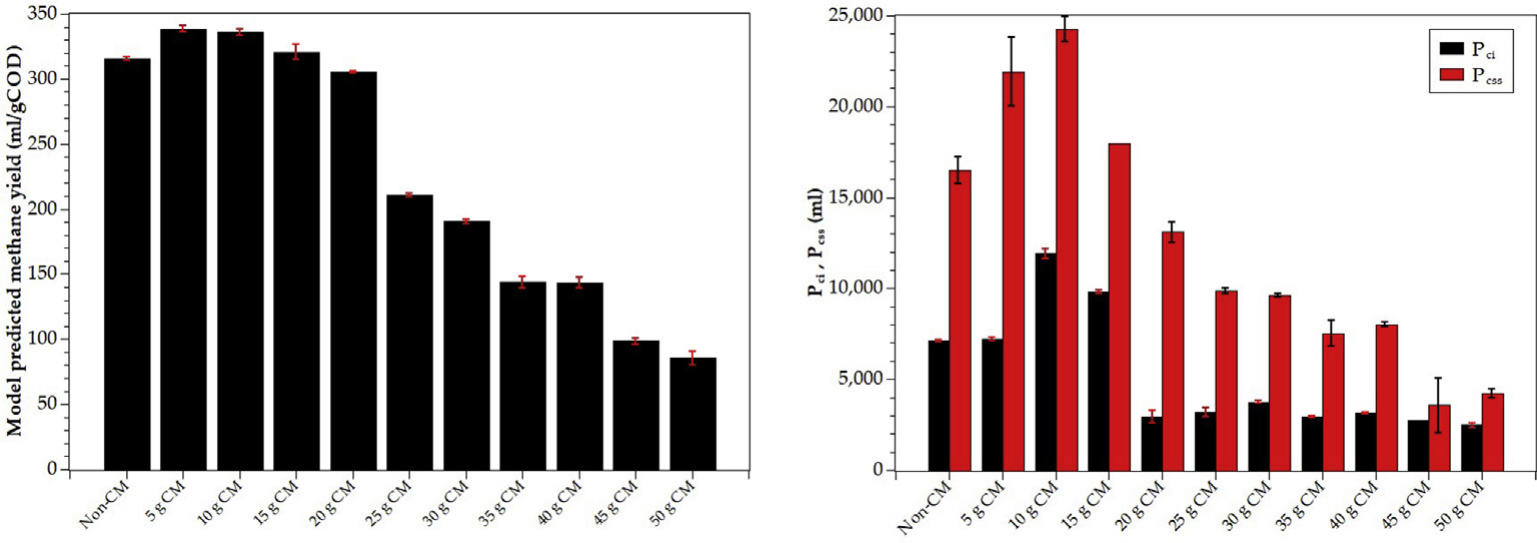
a) methane yield of the co-digestion of POME and chicken manure compared with the theoretical value (350 ml/gCOD) b) the first and the second critical accumulative methane ($P_{ci}$ and $P_{css}$ ) at different mixing ratios (or levels of CM supplement).

1. The non-degradable COD of the co-substrates was approximately 10–15% of the total COD by model fitting. By choosing 12% ($f_{n}\;=\;0.12$) for all mixing ratios, we obtained a very high correlation (R^2^ > 0.99). Adjusting $f_{n}$ within 0.1–0.15 did not improve the correlation significantly (up the fourth decimal point).
2. The results show that (Table 5), the easily degradable (ED), slowly degradable (SD), and very slowly degradable (VSD) COD were in the ranges of 23.7–44.8%, 35.0–53.6%, and 6.5–25.0%, respectively.
3. If we define *initial methanogenic activity (IMA)* as the product of $X_{0}\;and\;\mu_{me}.\;$ Without any CM supplement, the IMA was 1,055 mgCOD/d and dropped to 712.6 mgCOD/d by a 5g-CM supplement. Further increase in CM supplement reduced the IMA down to the range of 70–200 mgCOD/d. Thus, it is evident that CM supplement slowed down the initial methanogenic activity, probably due to ammonia toxicity. Although the methane yield was approximately constant for the CM-supplement range of 0–10g CM/40 ml POME, the addition of CM slowed down the initial methanogenic activity, requiring more time to reach the final methane potential.
4. The best-estimate of $K_{se}$ is in the range of 15,000–53,000 mg COD/l. The average value is 26,400 mg COD/l. This value is typical for the anaerobic digestion of POME and similar substrates.
5. The methane yield coefficients ($Y_{PSe}$) were close to the theoretical value (350 ml CH_4_/gCOD) for mixing ratios 0–20 g CM/40 ml POME. Further increase in CM supplement reduced $Y_{PSe}$ gradually (Table 5 and Figure 10a).
6. Generally, all seven parameters passed the significant tests at a 95% confidence level (standard error is within 50% limit).

**Table 5:** Parameters of the multi-substrate models obtained of fitting the model to the experimental ABE data from the co-digestion of POME and chicken manure.

Parameters	Chicken Manure										
	Non-CM	5 g CM	10 g CM	15 g CM	20 g CM	25 g CM	30 g CM	35 g CM	40 g CM	45 g CM	50 g CM
$f_{n}$	0.12	0.12	0.12	0.12	0.12	0.12	0.12	0.12	0.12	0.12	0.12
$f_{Sss}$	0.13	0.08	0.065	0.16	0.25	0.25	0.24	0.18	0.18	0.18	0.18
$f_{Ss}$	0.4725	0.536	0.4075	0.3708	0.378	0.378	0.4032	0.441	0.441	0.378	0.35
$f_{Se}$	0.2775	0.264	0.4075	0.3492	0.252	0.252	0.2368	0.259	0.259	0.322	0.35
$S_{0}\left(\text{mgCOD}\right)$	80,640	87,760	92,800	89,920	85,440	85,200	89,600	86,240	91,160	93,120	92,480
$X_{0}\left(\text{mgCOD}\right)$	2,000	2,000	400	200	200	200	200	500	400	700	700
Initial methanogenic activity $X_{0}\mu_{me}$ (mgCOD/d)	1,055	712.6	120.2	73.5	168.9	200.4	75.4	110.1	120.2	109.4	182.4
$\mu_{me}\left(d^{-1}\right)$	0.5273	0.3563	0.3006	0.3676	0.8444	0.4498	0.3769	0.2202	0.3004	0.1563	0.2666
SE (%)	±13.24	±9.12	±6.81	±7.57	±3.82	±25.92	±11.32	±5.41	±14.22	±13.71	±18.66
$K_{Se}\left(\text{mg}/\text{l}\right)$	19,607	20,938	14638	19,964	52,320	23,313	20,666	19,527	26,979	19,727	53,125
SE (%)	±20.33	±15.03	±19.30	±17.24	±4.00	±41.43	±18.83	±13.47	±24.23	±25.89	±30.57
∗$Y_{PSe}\left(\text{ml}/\text{mgCOD}\right)$	0.3158	0.3386	0.3360	0.3205	0.3055	0.2107	0.1907	0.1439	0.1433	0.0986	0.0855
SE (%)	±0.50	±0.64	±0.74	±1.82	±0.30	±0.66	±0.91	±3.15	±2.83	±2.42	±6.08
$P_{ci}\left(\text{ml}/\text{l substrate}\right)$	7,118	7,196	11,918	9,799	2,937	3,184	3,755	2,956	3,144	2,752	2,490
$S_{ci}=S_{0}-P_{Ci}/Y_{PSe}$ (mgCOD/l)	58,100	66,508	57,330	59,346	75,826	70,088	69,909	65,703	69,220	65,210	63,287
SE (%)	±0.71	±1.37	±2.32	±1.00	±12.12	±8.11	±1.58	±1.83	±1.45	±1.67	±4.54
$P_{css}\left(\text{ml}/\text{l substrate}\right)$	16,499	21,925	24,270	18,000	13,100	9,880	9,616	7,534	8,030	3,572	4,231
$S_{css}=S_{0}-P_{css}/Y_{PSe}\;$ (mgCOD/l)	28,394	23,008	20,568	33,758	42,560	38,309	39,175	33,890	35,124	56,892	42,994
SE (%)	±4.48	±8.86	±2.82	-	±4.25	±1.37	±1.06	±9.25	±1.25	±41.87	±5.66
P (ml/gCOD_added_) (BMP, 55 days)	278.9	276.9	275.2	234.9	264.3	177.8	158.0	103.1	105.2	79.45	59.38
∗P_e_ (ml/gCOD_added_) (55 days)	88.8	86.33	134.5	111.0	78.12	52.92	45.47	37.04	37.04	32.20	28.00
∗P_i_ (ml/gCOD_added_) (55 days)	190.1	190.6	140.7	123.8	186.1	124.9	112.6	66.09	68.17	47.25	31.38
$f_{\mu_{se}}\left(\text{no unit}\right)$	0.3687	0.3693	0.8784	0.6299	0.5659	0.6752	0.6488	0.7149	0.5302	0.8702	1.2025
SE (%)	±3.59	±2.22	±20.40	±18.78	±2.13	±22.46	±13.8	±15.79	±3.98	±24.02	±22.47
$f_{\mu_{ie}}\left(\text{no unit}\right)$	0.6424	0.8557	0.2787	0.4165	1.4264	0.4499	0.4453	0.5038	0.8476	0.6643	0.7008
SE (%)	±7.72	±8.22	±7.09	±50.48	±15.84	±52.56	±15.94	±13.59	±8.53	±12.37	±14.22
$f_{\mu_{sse}}\left(\text{no unit}\right)$	0.1649	0.0652	0.0995	0.0600	0.5856	0.7646	0.7437	0.04	0.04	0.05	0.04
SE (%)	±17.09	±11.88	±46.96	-	±10.62	±35.11	±16.16	-	-	-	-
**Adjusted**$R^{2}$	**0.9976**	**0.9984**	**0.9984**	**0.9987**	**0.9988**	**0.9993**	**0.9993**	**0.9993**	**0.9977**	**0.9979**	**0.9966**

Remark: The model passes the statistical F-test at a %99 confidence level for all data sets.

Note that $P_{ci}$ and $P_{css}$ are two critical or inflection points of the accumulated methane curve that characterize the methanogens' substrate preference and availability. Figure 10b depicts both critical points graphically. Both critical points reached their peaks at 10g CM supplement, confirming the best methane yield and healthy environment for methanogens. These two points coincide with the critical substrate concentration $S_{ci}$ and $S_{css}$ (see Table 5), the concentration where the methanogens are the halfway switching from the easily degradable substrate to the intermediates. The intermediates are the particular group of easily degradable substrates derived from the SD and VSD substrates' hydrolysis. The results show that $S_{ci}$ falls in the range of 57,000–76,000 mgCOD/l, whereas $S_{css}$ falls in a wider range of 20,000–57,000 mgCOD/l.

### 4.5 Fitting (calibrating) multi-substrate monod model to BMP data: POME-Gly co-digestion

**Figure 11:**
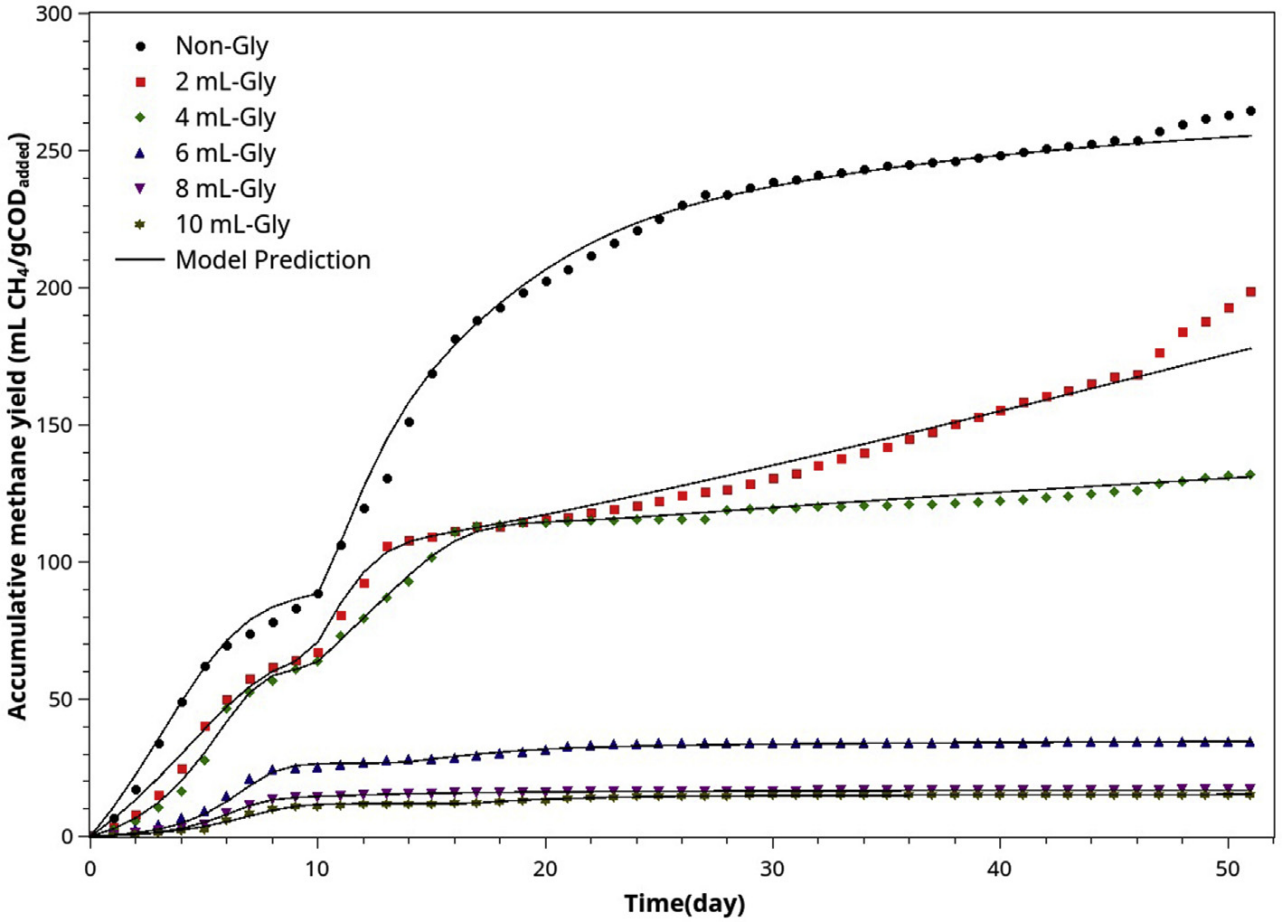
The comparison between experimental ABE curves (normalized) and the model's best-fit curves for the batch AcoD of POME with different Gly supplement levels.

Figure 11 compares the calibrated curves with POME-Gly AcoD data at six different ratios. Table 6 summarizes the parameters resulted from the model fitting. Although a similar strategy was used successfully for both cases, the POME-Gly AcoD differed significantly from POME-CM AcoD. Firstly, POME-Gly AcoD changes COD substantially. Even a small amount of Gly was added, high initial COD could result in the VFA accumulation, suppressing methanogenic activity and lowering the methane yields.

**Table 6:** Parameters of the multi-substrate models obtained from fitting the model to the experimental ABE data from the co-digestion of POME and crude glycerol.

Parameters	Crude glycerol					
	Non-Gly	2 mL-Gly	4 mL-Gly	6 mL-Gly	8 mL-Gly	10 mL-Gly
$f_{n}\left(\text{no unit}\right)$	0.15	0.0807	0.0528	0.03928	0.0313	0.02595
$f_{Sss}\left(\text{no unit}\right)$	0.09	0.5	0.5	0.8	0.85	0.86
$f_{Ss}\left(\text{no unit}\right)$	0.4712	0.1677	0.2147	0.0305	0.0119	0.0217
$f_{Se}\left(\text{no unit}\right)$	0.2888	0.2516	0.2325	0.1302	0.1068	0.0924
$S_{0}\left(\text{mgCOD}\right)$	82,000	152,380	232,761	313,142	393,523	473,904
$X_{0}\left(\text{mgCOD}\right)$	2,000	2,000	600	100	100	200
IMA $X_{0}\mu_{me}$ (mgCOD/d)	1,042	602	285	166.4	85.3	122
$\mu_{me}\left(d^{-1}\right)$	0.5211	0.3010	0.4753	1.6640	0.8531	0.6110
SE	±17.13%	±4.32%	±7.55%	±17.69%	±4.71%	±7.81%
$K_{Se}\left(\text{mg}/\text{l}\right)$	28186	19739	10129	59780	17250	15725
SE	±26.76%	±3.80%	±33.52%	±24.86%	±12.75%	±22.40%
$Y_{PSe}\left(\text{ml}/\text{mgCOD}\right)$	0.3141	0.2692	0.2639	0.2073	0.1336	0.1282
SE	±2.48%	±4.50%	±0.49%	±1.09%	±0.38%	±0.34%
$P_{ci}\left(\text{ml}\right)$	7272	10058	14388	8522	5643	5747
$S_{ci}=S_{0}-P_{ci}/Y_{PSe}$ (mgCOD/l)	58,848 71.0% of $S_{0}$	115,017 75.5%$\;of\;S_{0}$	178,240 76.6% of$\;S_{0}$	272,032 86.9% of $S_{0}$	351,285 89.3% of $S_{0}$	429,076 90.5% of $S_{0}$
SE	±2.03%	±3.67%	±0.59%	±0.95%	±0.43%	±0.35%
$P_{css}\left(\text{ml}\right)$	15000	16139	26833	10000	6361	10000
$S_{css}=S_{0}-P_{css}/Y_{PSe}\;$ (mgCOD/l)	34,245	92,428	131,082	264,902	545,910	395,000
SE	-	±10.75%	±0.78%	-	±0.90%	-
P (mlCH_4_/gCOD_added_) (BMP 51 days)	255.4	177.9	131.1	34.6469	16.90	15.25
Pe (mlCH_4_/gCOD_added_) (51 days)	89.24	67.93	61.46	26.6876	14.21	11.92
Pi (mlCH_4_/gCOD_added_) (51 days)	166.2	110.0	69.59	7.95931	2.691	3.329
$f_{\mu_{se}}$	0.3737	1.979	0.7113	2.538	3.674	2.9943
SE	±1.01%	±31.93%	±6.53%	±23.30%	±9.45%	±25.42%
$f_{\mu_{ie}}$	0.9406	0.9555	0.4521	0.7705	1	0.3449
SE	±11.45%	±32.00%	±25.93%	±107.90%	-	±21.02%
$f_{\mu_{sse}}$	0.0826	0.1826	0.02304	0.01	0.0872	0
SE	±45.09%	±21.68%	±11.42%	-	±30.83%	-
$\mathrm{Adjusted}\;R^{2}$	**0.9974**	**0.987**	**0.9973**	**0.9934**	**0.9986**	**0.9960**

Remark: The model passes the statistical test at a %99 confidence level for all data sets.

After calibration, the following observations are noted:

1. Gly is an easily degradable substrate. The best-fit fraction of SD substrate decreased by increasing Gly. Most of the slowly degradable substrates were derived from POME.
2. The increase of non-degradable substrate portion for higher Gly supplement means that the residue Gly was not further consumed because of the unfavorable environment due to VFA over-accumulation.
3. The methane yield coefficient ($Y_{PSe}$) dropped markedly as Gly supplement increased. It implied that the methanogenesis occurred at a sub-optimal condition.

According to Viana et al. [48, 49], Gly is readily converted to VFAs. Hence, the rate-limiting step can be either acetogenesis and methanogenesis. During the AD of Gly, the VFAs were consumed by acetogens and methanogens at a slower rate than acidogenic bacteria produce them. If the Gly supplement is too high, the over-produced VFAs, without a counterbalance by alkalinity, will inhibit the methanogenic activities, leading to the collapse of AD systems regardless of pH value [50].

Thus, POME-Gly co-digestion is not suitable for batch AD because Gly has a very high COD. It is readily degradable, resulting in too high VFA in a short period, which inhibits methane-producing bacteria. For the same reasons, theoretically, high COD makes Gly suitable for fed-batch and the completely mixed digestion system (CSTR digester).

Crude glycerol has a very high COD (900–1,230 gCOD/l). The theoretical methane composition of biogas obtained from glucose and glycerol is 67% and 72%, respectively. The experimental composition of biogas obtained from the AD of POME (64.0 %CH_4_) was close to the theoretical one (Figure 8a). Adding 2g of Gly to 40 ml of POME increased methane from 838 ml to 1,138 ml (36% increase in methane produced). However, the %methane in biogas dropped from 64% to 54.5%, and the BMP decreased from 255.4 mlCH_4_/gCOD_added_ to 177.9 mlCH_4_/gCOD_added_. Furthermore, the COD removal also dropped from 76.1% to 58.7%, and the initial methanogenic activity (IMA) reduced from 1,042 to 602 mgCOD/d (see Table 6). Our results agreed with Nuchdang and Phalakornkule [51] and Panpong et al. [52].

### 4.6 Model prediction of BMP/batch co-digestion tests and their potential applications

This section illustrates how to use the calibrated models to predict the state variables in batch co-digestion, which is not observable otherwise. We will use the data from three co-digestion cases for illustration: POME single digestion, POME-CM co-digestion (5 gCM added into 40 mlPOME), and POME-Gly co-digestion (2 mlGly added into 40 mlPOME).

#### 4.6.1 POME single digestion

**Figure 12:**
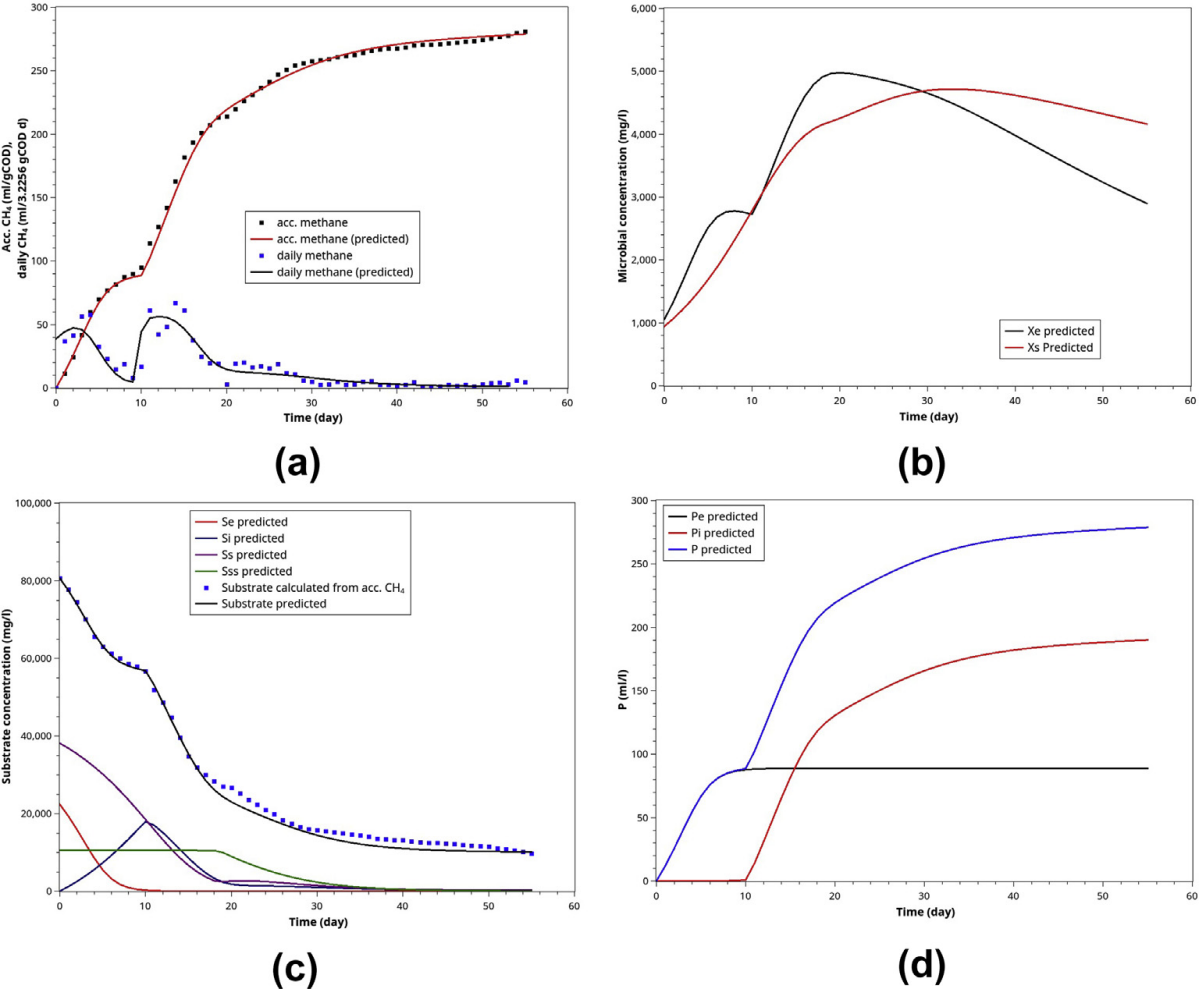
Model simulation for POME single anaerobic digestion: (a) the normalized ABE curves and daily methane (based on 3.9492 initial gCOD), (b) the microbial activities of methanogens ($X_{e}$) and hydrolytic bacteria ($X_{s}$), (c) the substrates' profile during the batch AD, (d) the methane contributed by the ED substrates and those derived from SD and VSD substrates.

POME has a well-balanced ED and SD substrate, which helps to synchronize hydrolysis and acidogenesis, ensuring a high methane yield ($Y_{PSe}$ = 0.314–0.316 ml/mgCOD). Moreover, POME single batch-AD exhibits consistent and repeatable ABE curves where the methanogens switch from ED to intermediates (from hydrolysis of SD and VSD substrates) was observed (Figures 5, 7, 9, 11, and 12). Alternatively, The daily methane curves exhibited two distinct peaks (Figure 12a). Other predictions in Figure 12b,c,d are intuitive and self-explanatory.

#### 4.6.2 POME-CM co-digestion (5 gCM added into 40 mlPOME)

**Figure 13:**
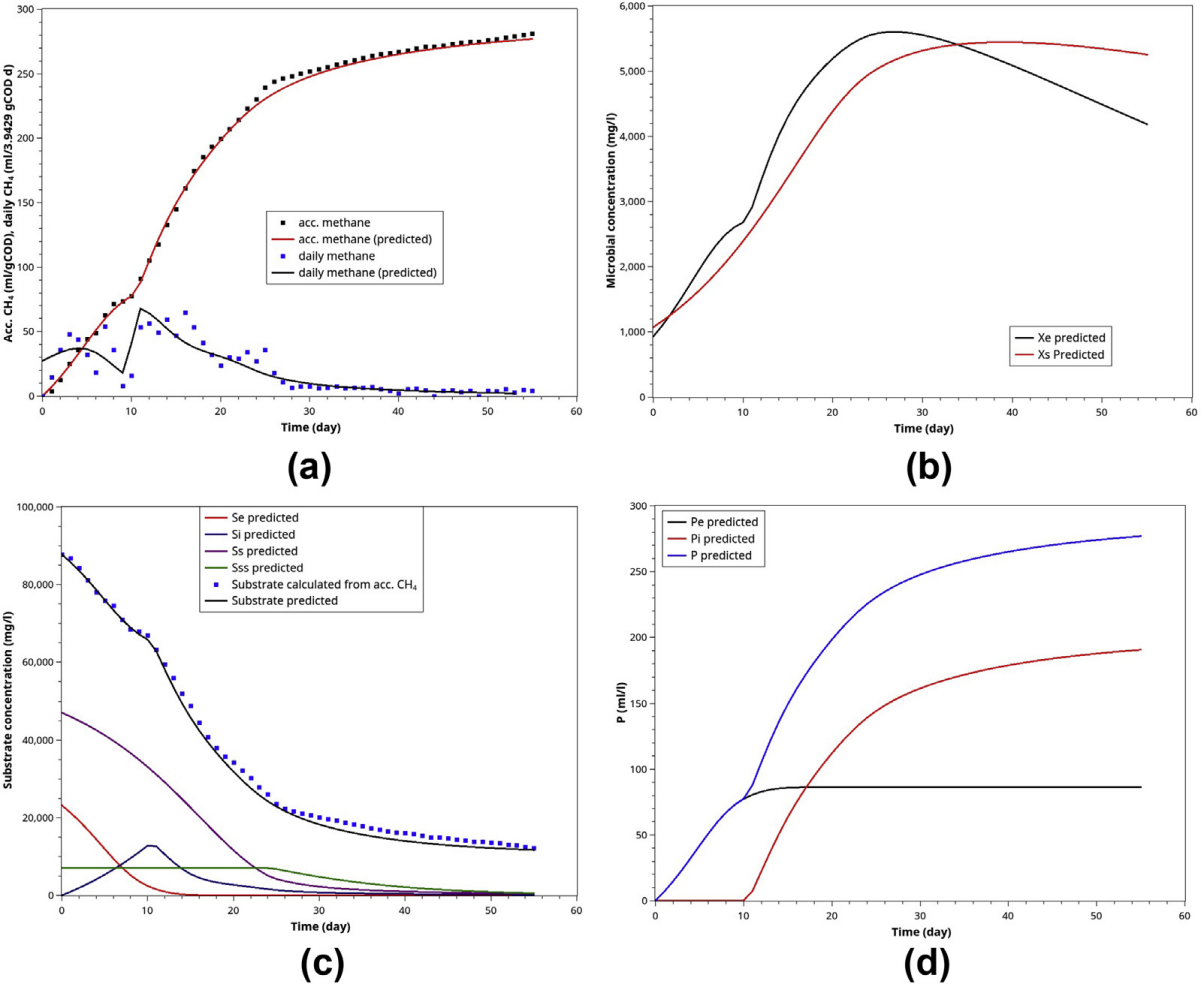
Model simulation for AcoD of 40 ml POME co-digested with 5 g CM: (a) the normalized ABE curves and daily methane (based on 3.9492 initial gCOD), (b) the microbial activities of methanogens ($X_{e}$) and hydrolytic bacteria ($X_{s}$), (c) the substrates' profile during the batch AD, (d) the methane contributed by the ED substrates and those derived from SD and VSD substrates.

As shown in Figure 13, when 5g of CM was added into 40 ml of POME, the model simulation agrees well with experimental data in a very similar manner to the mono-digestion of POME (Figure 12). At this level of CM supplement (0–20 g CM), the methane yield coefficients were close to the theoretical value (0.35 ml/mgCOD) in the range of 0.306–0.338 ml/mgCOD, indicating a well-balanced environment for AcoD. Further increase in CM supplement adversely affected the methane yield due to the ammonia accumulation.

The behavior of POME-CM AcoD leads us to conclude that at the low level of CM supplement, CM has an additive effect on the methane yield but not necessarily a synergistic one.

#### 4.6.3 POME-Gly co-digestion (2 mlGly added into 40 mlPOME)

**Figure 14:**
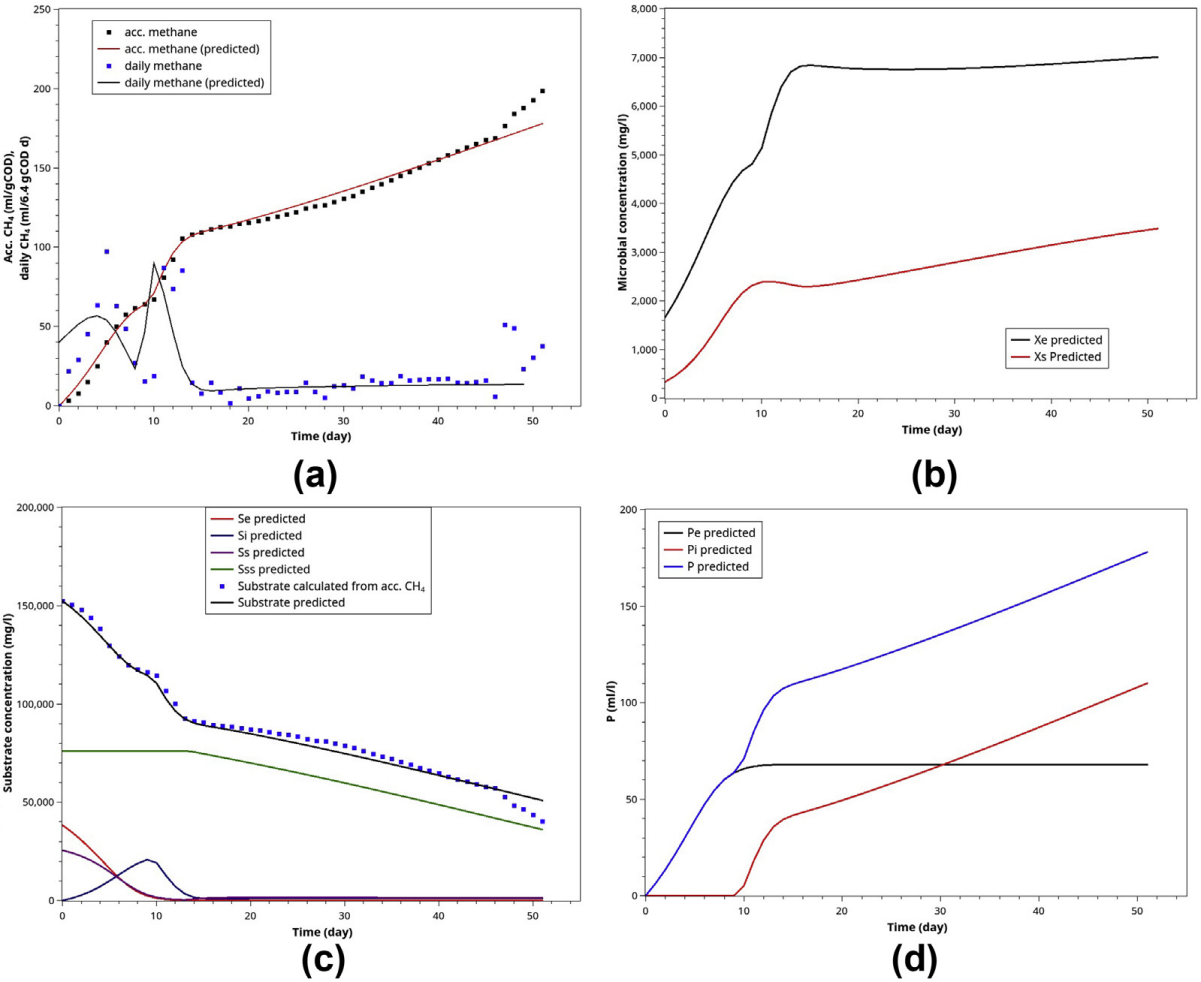
Model simulation for AcoD of 40 ml POME co-digested with 2 ml Gly: (a) the normalized ABE curves and daily methane (based on 6.4 initial gCOD), (b) the microbial activities of methanogens ($X_{e}$) and hydrolytic bacteria ($X_{s}$), (c) the substrates' profile during the batch AD, (d) the methane contributed by the ED substrates and those derived from SD and VSD substrates.

The behavior of POME-Gly AcoD differed from that of POME-CM significantly. Adding a large amount of Gly (>4ml/40 mlPOME) into POME inhibited methane production almost entirely. At a mild level, 2 ml Gly (mixed into 40 mlPOME), the experimental data and the prediction are shown in Figure 14. Although Gly is considered an ED substrate, it is evident that the methanogens produced CH_4_ from the ED portion of POME in the first ten days of digestion. The methanogens would start to convert Gly into VFA and then methane. The ABE curves show that after day 12 onwards, CH_4_ increased at a much slower pace than the first 12 days. It could be explained as a parallel conversion of Gly to VFA and VFA to CH_4_, where methanogenic reactions were the rate-limiting ones.

The results in our series of batch tests show that in POME-Gly AcoD, the concentration of Gly in the digestate should not exceed 5%, preferably less than that [53]. This Gly level can be controlled easily in an industrial CSTR or fed-batch digesters. It should also be noted that the extra methane obtained by Gly supplement was not derived from any synergistic effect but purely from the COD added by Gly itself. This fact is also confirmed by other authors [48, 53].

### 4.7 POME anaerobic in 14-L CSTR: model calibration

**Figure 15:**
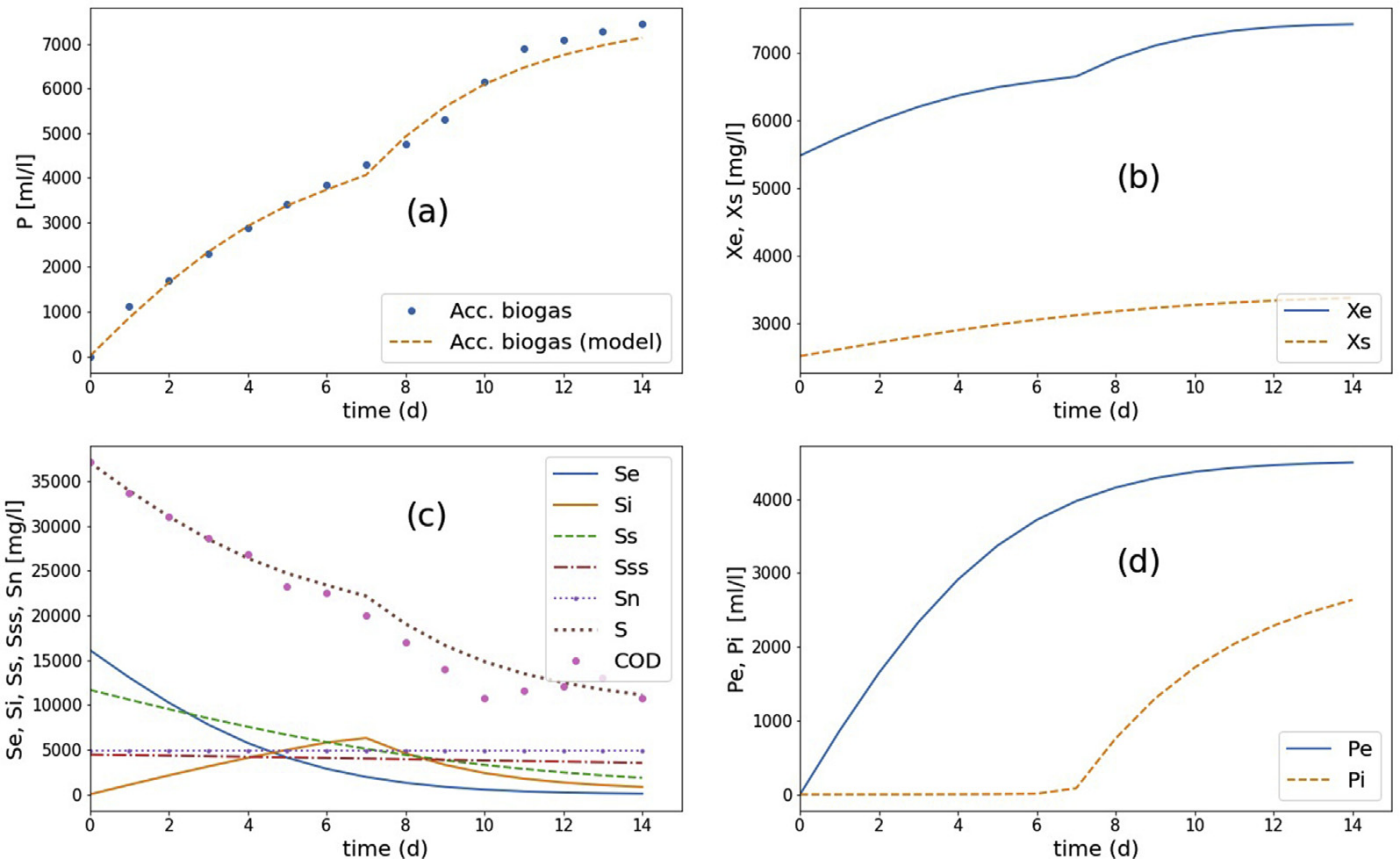
The results of AD CSTR experiments and the model simulation, comparison between the experiment and the simulation of the calibrated model for start-up period: (a) the accumulated biogas generation, (b) microbial activities, (c) substrate conversion, and (d) biogas contribution from the easily degradable substrate ($P_{e}$) and slowly degradable substrate ($P_{i}$).

The 14-L AD CSTR was carried out into two periods: start-up (batch AD for days 0–14) and semi-continuous periods (days 15–45). The process was stable for prolonged operation for more than three months. After filling the reactor with POME (7 L) and AD sludge (7 L), the calculated COD was 37,120 mgCOD/l. The experimental results, including accumulated biogas and the average COD, were shown in Figure 15.

In the simulation, the following parameters were set to the calibrated values (R^2^ > 0.99): $S_{0}=\;37,120\;mgCOD/l$, $f_{n}\;=\;0.13,\;f_{Sss}=0.12,$ $f_{Ss}=0.42,\;\mu_{me}=\mu_{ms}=0.13$ d^−1^, $\mu_{mi}=\;0.17$ d^−1^, $\mu_{mss}=\;0.013$ d^−1^ $,K_{Se}=\;K_{Si}$ $=K_{Ss}$ $=K_{Sss}$ = 20,000 mgCOD/l, $X_{0}$ = 8,000 mg/l, $X_{s}=$ 3,360 mg/l, $X_{e}=$4,640 mg/l, $Y_{PSe}=Y_{PSi}\;=\;0.28\;$mlCH_4_/mgCOD, κ=0.1, $P_{c}=$4,000 ml. These calibrated parameters, except $\mu_{me},\mu_{ms},\mu_{mi}$ and $\mu_{mss}$ are approximately the same magnitudes as those obtained in BMP tests. This result shows that the BMP parameters could predict the start-up period of AD CSTR operation. However, the microbial activity can be varied depending on the amount of AD sludge used in the start-up and how active the microbes in the sludge.

**Figure 16:**
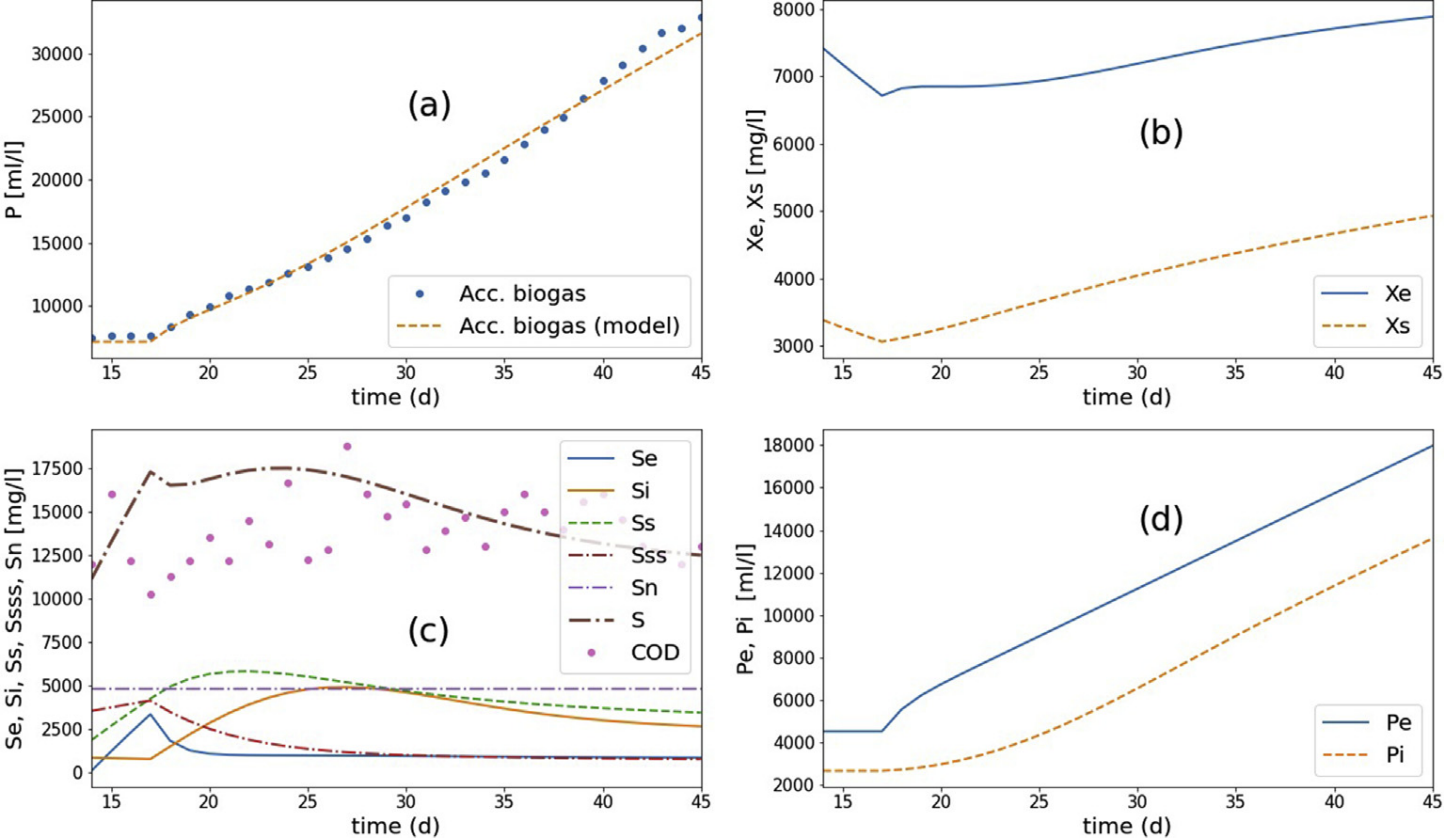
The results of AD CSTR experiments and the model simulation, comparison between the experiment and the simulation of the calibrated model after switching from start-up to continuous period: (a) the accumulated biogas generation, (b) microbial activities, (c) substrate conversion, and (d) biogas contribution from the easily degradable substrate ($P_{e}$) and slowly degradable substrate ($P_{i}$).

After switching into the semi-continuous mode ($S_{in}=\;81,769$ mgCOD/l) for 30 days, it was found that the calibrated values of a few parameters changed but within the normal range observed in BMP tests. These values are: $\mu_{me}=0.356,\;\mu_{ms}=0.178$ d^−1^, $\mu_{mi}=\;0.16$ d^−1^, $\mu_{mss}=\;0.013$ d^−1^, $Y_{PSe}=Y_{PSi}\;=\;0.25\;$mlCH_4_/mgCOD. The results are shown in Figure 16. There was a 2–3 days lag after switching to semi-continuous mode before the biogas start to appear again. During the lag, the model predicted that the nutrient consumption ceased temporarily. However, the experimental result showed that the carbon source (COD) was still being consumed continuously. Hence, the current model does not describe the lag mechanistically.

We have shown that the multi-substrate Monod model's parameters obtained from BMP tests in the start-up and continuous AD operation of POME are similar. The discrepancies were due to various biochemical and environmental factors, including flow arrangement and variations in sludge activity and wastewater components.

## 5 Conclusion

POME is a well-balanced substrate for the AD process, containing sufficient macro and micronutrients. Its C/N ratio is also in the optimal range. This fact was confirmed by near-theoretical methane yield co-efficient ($Y_{PSe}$=0.314 ml/mgCOD). Supplementing CM in the range of 0–50%wt (add CM up to 20g to 40 ml POME) did not change the methane yield co-efficient significantly but gradually slow down the methanogenic activities. The reduced methane production rate could be attributed to free ammonia accumulation.

On the other hand, Gly is a high-COD substrate. Supplementing Gly only 5% (adding 2ml of Gly into 40 ml of POME) almost double the total COD. As Gly is easily degradable, this, in turn, the acidogens can readily convert it to VFAs. Over-accumulation of VFA could suppress the methanogenic activity, causing low methane yield ($Y_{PSe}$=0.269 ml/mgCOD). This suppression may not show up strongly for the continuous AD process because of the dilution effect in CSTR reactors. Furthermore, there no evidence that Gly has any synergistic effect on co-digestion, but its contribution to the increase in biogas/methane was due to its own COD.

POME can serve as an excellent platform for co-digestion. Since it is self-sufficient in nutrients, non-toxic, and can be degraded readily, co-digestion could not significantly improve the yield synergistically. However, the co-digestion of POME with CM and Gly can generate on-demand biogas generation responding to the electricity load of the factories or the grid lines.

One of the article's main contributions is developing a simple multi-substrate in line with AMD1 and AMOCO. The proposed model was able to describe the BMP and batch AD data very well. A limited CSTR experiment illustrated its extension for a continuous AD operation. However, more extensive validation would be required before industrial applications can be practically realized.

## Declarations

### Author contribution statement

Narongsak Seekao: Performed the experiments; Analyzed and interpreted the data.

Sawinee Sangsri: Performed the experiments.

Nirattisai Rakmak & Wipawee Dechapanya: Analyzed and interpreted the data.

Chairat Siripatana: Conceived and designed the experiments; Analyzed and interpreted the data; Wrote the paper.

### Funding statement

This work was supported by Walailak University Fund (Contact no. WU62217).

### Data availability statement

Data will be made available on request.

### Declaration of interests statement

The authors declare no conflict of interest.

### Additional information

No additional information is available for this paper.
